# Sequence search and analysis of gene products containing RNA recognition motifs in the human genome

**DOI:** 10.1186/1471-2164-15-1159

**Published:** 2014-12-22

**Authors:** Sony Malhotra, Ramanathan Sowdhamini

**Affiliations:** National Centre for Biological Sciences (TIFR), GKVK Campus, Bellary Road, Bangalore, 560 065 India

**Keywords:** RNA recognition motif, *Homo sapiens*, Genome-wide survey, Domain architecture, Splicing

## Abstract

**Background:**

Gene expression is tightly regulated at both transcriptional and post-transcriptional levels. RNA-binding proteins are involved in post-transcriptional gene regulation events. They are involved in a variety of functions such as splicing, alternative splicing, nuclear import and export of mRNA, RNA stability and translation. There are several well-characterized RNA-binding motifs present in a whole genome, such as RNA recognition motif (RRM), KH domain, zinc-fingers *etc*. In the present study, we have investigated human genome for the presence of RRM-containing gene products starting from RRM domains in the Pfam (Protein family database) repository.

**Results:**

In Pfam, seven families are recorded to contain RRM-containing proteins. We studied these families for their taxonomic representation, sequence features (identity, length, phylogeny) and structural properties (mapping conservation on the structures). We then examined the presence of RRM-containing gene products in *Homo sapiens* genome and identified 928 RRM-containing gene products. These were studied for their predicted domain architectures, biological processes, involvement in pathways, disease relevance and disorder content. RRM domains were observed to occur multiple times in a single polypeptide. However, there are 56 other co-existing domains involved in different regulatory functions. Further, functional enrichment analysis revealed that RRM-containing gene products are mainly involved in biological functions such as mRNA splicing and its regulation.

**Conclusions:**

Our sequence analysis identified RRM-containing gene products in the human genome and provides insights into their domain architectures and biological functions. Since mRNA splicing and gene regulation are important in the cellular machinery, this analysis provides an early overview of genes that carry out these functions.

**Electronic supplementary material:**

The online version of this article (doi:10.1186/1471-2164-15-1159) contains supplementary material, which is available to authorized users.

## Background

The gene expression process in eukaryotes needs to be tightly regulated at every step. Firstly, it is regulated at the transcription level by means such as chromatin structure, DNA sequence elements and binding of transcription factors *etc.* [1, 2]. In spite of this tight regulation, post-transcriptional regulation plays an important role in regulating the levels of mRNA that are expressed in all tissues and serves as a supplement control mechanism. The post-transcriptional regulation governs several processes namely alternative splicing, RNA editing, transport of RNA from nucleus to cytoplasm, RNA stability and translation [1, 3]. The aberrations in the regulation of gene expression are also implicated in several human diseases such as Huntington’s disease, leukoencephalopathy, cancer *etc.* [4–6].

RNA-binding proteins (RBPs) mediate all the post-transcriptional control events. As there are varied levels of control and targets to be regulated, there exists a wide repertoire of RNA-binding motifs. To achieve the sequence-specific recognition of targets, there are several RNA-binding domains that are well-characterized in RBPs such as RRM (RNA recognition motif) domains, KH domains, pumilio homology domain, zinc fingers, double-stranded RNA binding motifs (dsRBMs) [7, 8]. RRM is the most abundant RNA-binding domain in higher vertebrates and is also known as RNA binding domain (RBD) or ribonucleoprotein domain (RNP) [9].

RRM is ~80-90 amino acids in length and contain two conserved motifs, RNP1 and RNP2, which are rich in aromatic amino acids. RRM structure possesses β_1_α_1_β_2_β_3_α_2_β_4_ topology, containing a four-stranded β –sheet which is packed against two α-helices [7, 9]. RNP1 is eight amino acids long and is present on β_3_ and RNP2 (six amino acids) is present on β_1_ [9]. Recently, RRM domains are also reported to be associated with the RNA-binding prion candidate proteins [10]. Birney *et al.*, performed an analysis on 125 sequences (possessing 252 RRM) of splicing factors and reported three solvent-exposed aromatic conserved residues in RNP-1 and RNP-2, which are implicated in RNA-binding [11].

Detailed study of RRM domains and their functions in the available sequenced genomes will help to improve our understanding and functions of RBPs. RBPs can be identified by the identification of RNA-binding domains in a given genome of interest. There have been several attempts to perform genome-wide analysis for specific RBPs in various organisms such as in *Drosophila melanogaster*, *Mus musculus*, *Arabidopsis thaliana*, sponge *Amphimedon queenslandica*, *C.elegans,* and yeast genomes [12–16]. This has led to identification of several RBPs in these genomes and 5-8% of genes are reported to encode RBP in yeast and ~2% in *C. elegans, D. melanogaster* and mouse. These studies provide insight into the distribution of RBPs and their classes in the genome being examined and their underlying functions.

In the present study, we have performed sequence searches in the human genome. We first studied the RRM-containing protein families in the Pfam database [17–20] for their taxonomic distribution, sequence features (sequence identity, phylogeny) and mapped conserved residues on their structures. We employed the profiles built using the members of these families to perform searches in the *Homo sapiens* genome. The gene products that retain sequence signatures of RRM-domains were next studied for their domain architectures, biological processes, pathways and disease relevance.

## Results and discussion

### RRM families in Pfam

**Figure 1:**
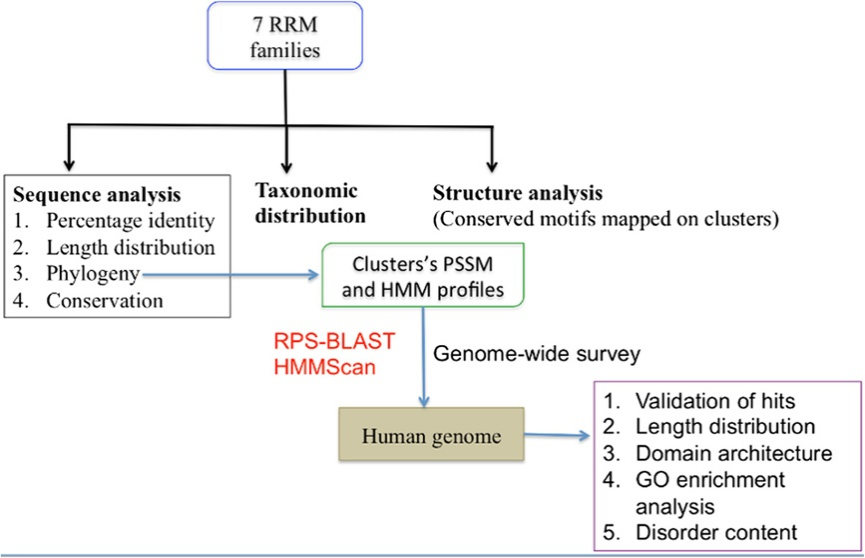
**Overall schema of the methodology and analysis.** The figure highlights the analysis performed on RRM-containing Pfam families and the methodology adopted to perform genome-wide survey in *Homo sapiens* genome.

There are seven families in the protein sequence family database (Pfam), which possess RRM domains (Table 1). We studied these families for their sequence features, taxonomic distributions and structural features (Figure 1).

**Table 1:** **RRM families in Pfam database**

Pfam ID	Pfam name	Description	Number of Pfam seed sequences
PF00076	RRM_1	Clan RRM, has splicing factor and GO annotation as nucleic acid binding. Well-characterised	79
PF04059	RRM_2	Clan RRM, Meiosis protein mei2	4* (310)
PF08777	RRM_3	Clan RRM, La protein (RNA chaperone), 5 stranded Beta sheet (atypical RRM)	15* (185)
PF10598	RRM_4	PrP8 protein (Large RNA protein complex of spliceosome)	25* (233)
PF13893	RRM_5	Clan RRM, hnRNP	107
PF14259	RRM_6	Clan RRM	79
PF10378	RRM	Found only in fungi, putative RNA binding domain	7* (74)

There are seven families defined in Pfam based on their gathering threshold values. The families are listed with their description and the number of seed sequences in Pfam.

*The numbers in brackets represent the full set members when the seed sequences are less than 50 in number.

**Figure 2:**
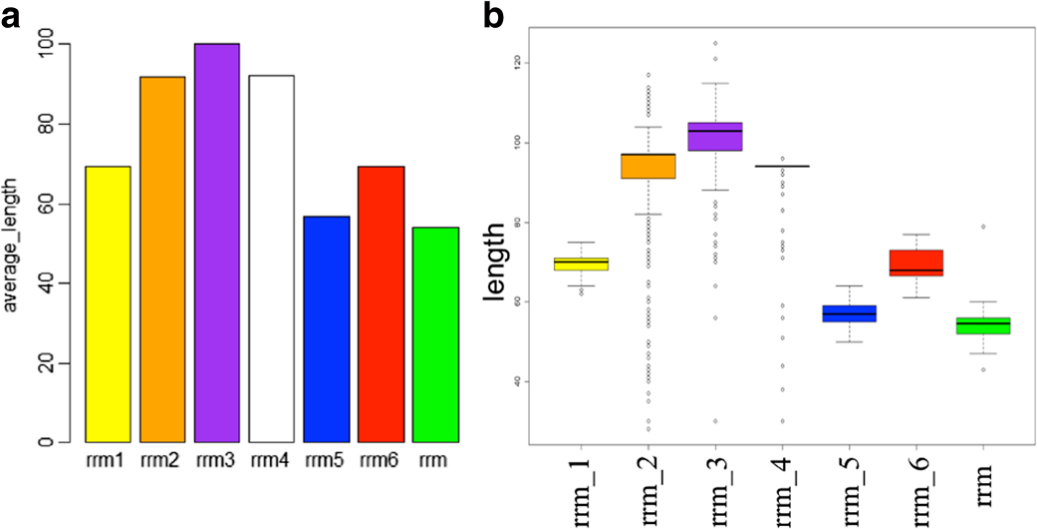
**Length distribution within RRM family members in Pfam database.** We studied the protein length distribution of RRM families’ members. **(a)** Average amino acid length of a RRM family and **(b)** The distribution of amino acid lengths of the members of the seven RRM families in Pfam.

RRM domains are known to be ~80-100 amino aids in their length [9] and Figure 2 shows the length distribution of the members for the seven RRM families in Pfam database. The proteins possessing RRM domains are present mainly in Eukaryota (Metazoa, Viridiplantae, Fungi kingdoms). But there is very little representation of RRM_1 domain in Bacteria (Additional file 1).

#### (i) Sequence features

We examined the sequence dispersion of members within and across the seven families for the distribution of sequence identities among and across family members and domain lengths.

**Figure 3:**
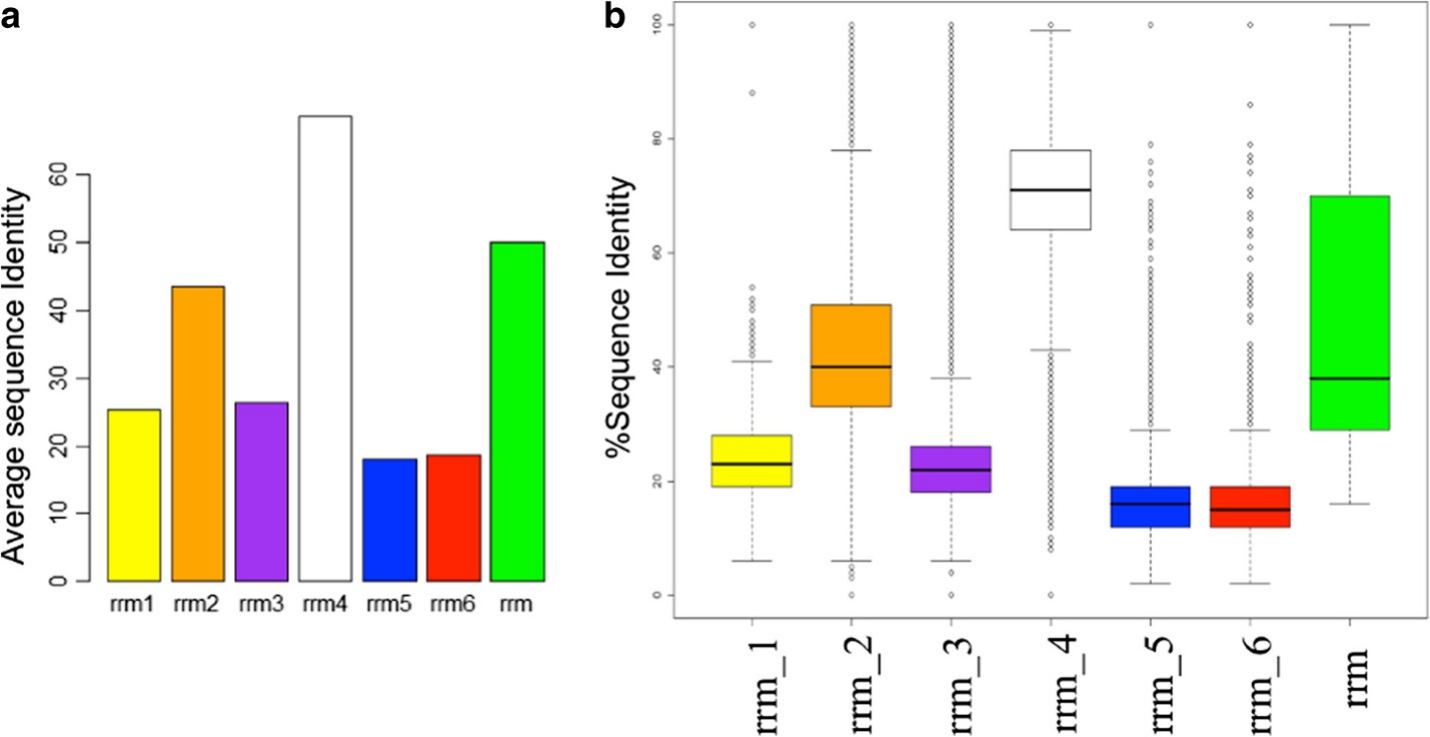
**Sequence identity within RRM families’ members.** We studied the sequence diversity between the members within a RRM family. **(a)** It shows the average sequence identities of different families and **(b)** The distribution of percentage sequence identity of the seven RRM families in Pfam.

RRM domains from the members of the same families were observed to be very diverse at the sequence level (Figure 3). The average sequence identity (Figure 3A) of the seven RRM families was <40% for four families (RRM_1, RRM_3, RRM_5 and RRM_6). The distribution of sequence identities between members of the same family is plotted in form of a Box-Whisker plot (Figure 3B). Across the different RRM families, as expected, the average percentage identity was <20%. However, across families of RRM_1, RRM_5 and RRM_6, some members share high sequence identity, as shown in Additional file 2.

The Pfam seed sequences from all the seven RRM families were employed (full set if seed set contains <50 sequences, Table 1) to generate phylogeny using neighbor-joining method using ClustalW [21, 22], Additional file 3A) and maximum-likelihood (PhyML [23], Additional file 3B) method. Sequences from families RRM_1, RRM_5 and RRM_6 were observed to co-cluster, consistent with the observation of distribution of sequence identities (please see above). We refined the alignment of seed sequences using MUSCLE 3.8 [24] and built a new neighbor-joining tree using MEGA 6 [25] (Additional file 3C) using 500 bootstraps. The co-clustering of the members belonging to the three Pfam-defined families (RRM_1, RRM_5 and RRM_6) was still persistent. Therefore, we defined new distinct clusters derived from the phylogeny (Additional file 3C, inner circle). The sequences of these clusters were re-aligned using MUSCLE 3.8 [24] and their PSSM and HMM profiles were created (please see Methods for details) to perform the searches in the human genome.

#### (ii) Structural features

**Figure 4:**
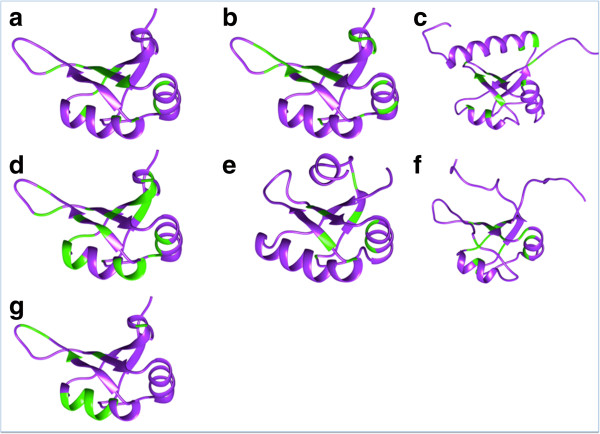
**Conservation mapping on RRM structures.** The multiple sequence alignments for each of these families were employed to study sequence conservation and the conserved residues were mapped to the RRM structures (in green). However for the families RRM_2, RRM_4 and family-RRM, there is no structural representation and we therefore, used structure of RRM_1 family for mapping. **(a)** RRM_1 (1L3K, chainA), **(b)** RRM_2, **(c)** RRM_3 (1OWX, chainA), **(d)** RRM_4, **(e)** RRM_5 (1A9N, chain **d**), **(f)** RRM_6 (1WG5, chain **a)** and **(g)** RRM.

Four of the seven families have structural representation in the Protein Data Bank (PDB) [26]. The alignments for the seven families were analyzed for the conservation of residues using ConSurf [27]. The conserved residues were mapped on the structures from each of the Pfam RRM family (Figure 4). We observed that the conserved residues map to the same structural region. This observation, together with the percentage similarity plots (across families, Additional file 2) explains the cause of co-clustering observed between the members of the different RRM families. Therefore, as explained above, we made distinct clusters based on sequence identities to perform searches in the human genome.

### Searches in the human genome and validation

Both PSSM and HMM profiles of the new clusters (please see Methods for details) were employed to search the human genome for the presence of RRM-containing gene products using RPS-BLAST [28, 29] and HMMscan [30] respectively. 928 RRM-containing gene products were thus identified in the human genome purely by sequence searches (Additional file 4). Of these, 50% (452 gene products) are unreviewed proteins and belong to UniProt/TrEMBL. Subsequent to the clustering using BLASTCLUST [31, 32] (at 98% sequence identity), 403 human gene products were retained. 84% of these (340 gene products) are annotated in Gene Ontology (GO) database [33] for their molecular functions. Out of these, 337 gene products were annotated as RNA-binding (and child terms) and/or nucleotide binding (and child terms) in GO.

### Length distribution

**Figure 5:**
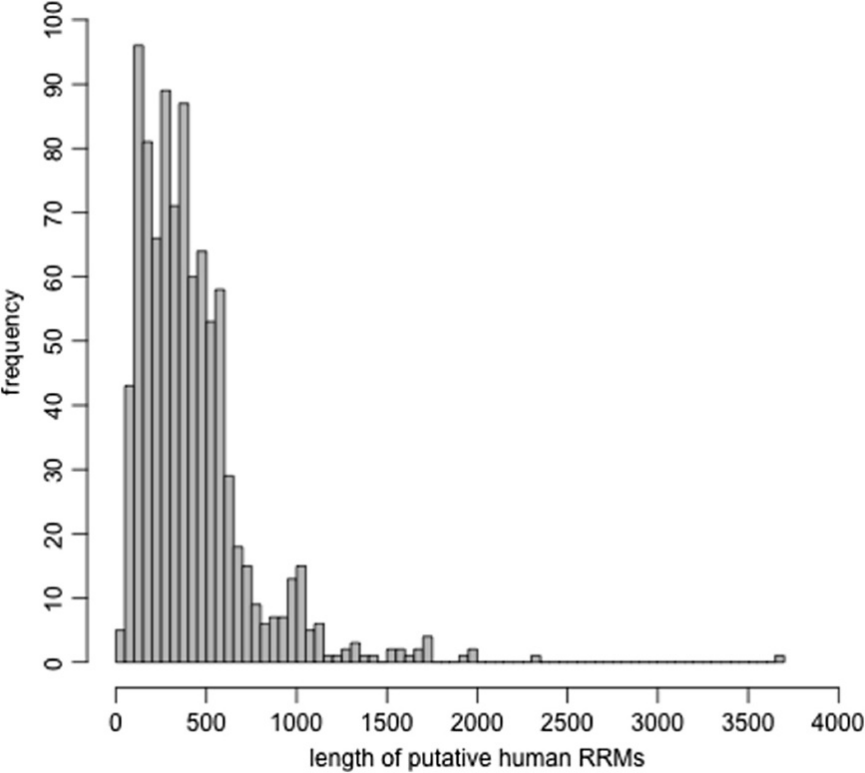
**Length distribution of the RRM-containing human gene products.** We studied the protein length distribution of the human gene products that were identified as RRM-containing.

The full-length distribution of the RRM-containing gene products identified by sequence searches was next analyzed. RRM is a small domain of ~80-100 amino acids; however, we observed that most of the RRM-containing human hits are >150 amino acids in length (Figure 5). This implies that there are other co-existing domains or multiple RRM domains or unstructured regions accompanying RRM domains in the full-length human gene products that contain RRM domains.

### Domain architectures (co-existing domains)

The full-length RRM-containing human gene products were also analyzed for their complete domain architectures using HMMScan [30] against HMM profiles of Pfam families. Such a search enabled the association of RRM domains identified in the human genome into any one of the known seven families in Pfam database as well. RRM_1 (PF00076) is the most well-characterized and well-populated RRM family in the Pfam database. 79% of the identified human RRM-containing gene products possess RRM_1 domain. In Pfam database, RRM_1 is present in larger fraction of protein sequences of the class Mammalia as compared to other six RRM families (Additional file 1). Out of the seven RRM families in Pfam, we observed that two families (RRM_2 and RRM) have no representation in the human genome. There are no sequences from the class mammalia for these two families in the Pfam database also. RRM is a fungal-specific family and RRM_2 is found only in Viridiplantae and fungi (Additional file 1).

**Figure 6:**
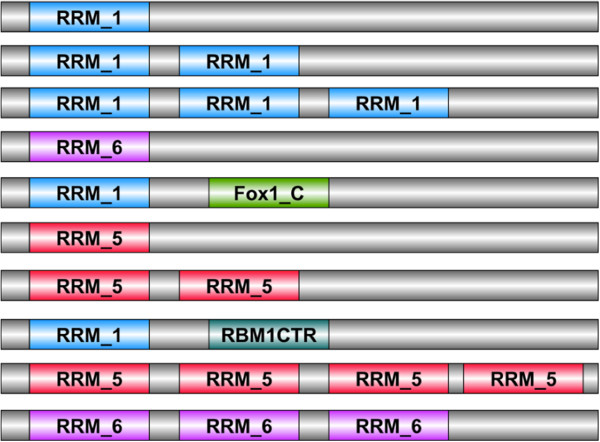
**Domain architectures of the RRM-containing human gene products.** We studied the human gene products for the presence of the co-existing domains. The schematic diagram displays the most frequent domain architectures drawn using the software DOG 1.0.

We further analyzed the co-existing domains and observed that in 13 gene products (where isoforms are reported), the number of RRM occurrence and co-existing domains are different (Table 2). This implies that may be during the alternative splicing event full domains are also spliced out. Figure 6 highlights the ten most frequent domain architectures observed in RRM-containing human gene products in a schematic form drawn. 40% of the gene products contain no other recognizable co-existing domain, whereas rest of the sequences possesses either multiple RRM domains or other co-existing domains. The gene products containing a single domain connect to RRM_1/RRM_5/RRM_families and map to their biological processes using GO annotations. 39 of these gene products are annotated with their biological process (Additional file 5).

**Table 2:** **Domain architectures in isoforms**

Domain architecture	Human protein	Domain architecture	Human protein
RRM_1,RRM_1,RRM_1	O60506	PWI, RRM_5,RRM_5	Q5T8P6
RRM_1,RRM_1	O60506-2	PWI, RRM_5,RRM_5	Q5T8P6-2
RRM_1,RRM_1,RRM_1	O60506-3	PWI, RRM_5,RRM_5	Q5T8P6-3
RRM_1,RRM_1	O60506-4	RRM_5,RRM_5	Q5T8P6-4
RRM_1,RRM_1,RRM_1	O60506-5	RRM_5,RRM_5	Q5T8P6-5
La, RRM_1,RRM_3	Q4G0J3	RRM_1,RRM_1	Q86SG3
RRM_3	Q4G0J3-2	RRM_1	Q86SG3-2
RRM_5	O95628	RRM_1,RRM_1,RRM_1,RRM_1	Q8IUH3
RRM_5	O95628-2	RRM_1,RRM_1,RRM_1	Q8IUH3-2
RRM_5	O95628-3	RRM_1,RRM_1,RRM_1,RRM_1	Q8IUH3-3
zf-C3HC4_3,RRM_5	O95628-4	RRM_1,RRM_1,RRM_1	Q8N6W0
RRM_5	O95628-5	RRM_1,RRM_1	Q8N6W0-2
RRM_5	O95628-6	zf-CCCH,zf-CCCH	Q8WU68
zf-C3HC4_3,RRM_5	O95628-7	zf-CCCH	Q8WU68-2
zf-C3HC4_3,RRM_5	O95628-8	zf-CCCH,zf-CCCH	Q8WU68-3
RRM_6,RRM_6	P31942	RRM_5,RRM_5,RRM_5	Q8WVV9
RRM_6,RRM_6	P31942-2	RRM_5,RRM_5	Q8WVV9-2
RRM_6,RRM_6	P31942-3	RRM_5,RRM_5	Q8WVV9-3
RRM_6	P31942-4	RRM_5,RRM_5,RRM_5	Q8WVV9-4
RRM_6	P31942-5	RRM_1,RRM_1,RRM_1	Q96J87
RRM_6	P31942-6	RRM_1,RRM_1	Q96J87-2
RRM_1,RRM_1,RRM_1	Q9P2K5	RRM_1,RRM_1,RRM_1	Q96J87-3
RRM_1,RRM_1,RRM_1	Q9P2K5-2	RRM_1,RRM_1	Q96J87-4
RRM_1	Q9P2K5-3		

928 RRM-containing gene products were studied for their co-existing domains. We observed that in 13 gene products where isoforms are reported the domain architectures are different. This implies may be during the alternative splicing event full domains are also spliced out.

**Figure 7:**
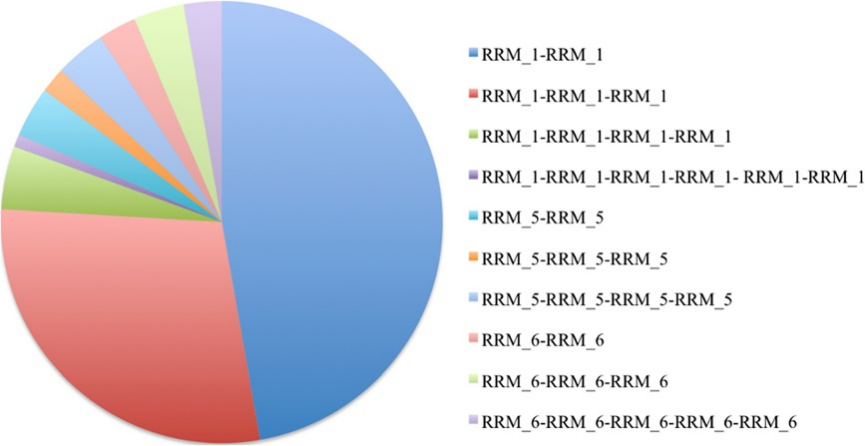
**Modular nature of RRM.** Within a single polypeptide sequence, RRM domain was observed to occur multiple times in 108 of the human gene products. This figure highlights the distribution of different RRM domains and their multiple occurrences. RRM_1 is present most frequently (88 of the gene products) and is repeated twice within a single protein sequence.

Genes containing multiple RRM domains were present in 28% of the RRM-containing human hits (Figure 7) and this event is a frequent occurrence almost as a rule [9, 34]. There are 56 non-RRM co-existing domains (Additional file 6) which were noted for their functions and are observed to be involved in a variety of cellular activities such as developmental signaling, apoptosis, transcriptional regulation, splicing and alternative splicing (Additional file 6). We also noted these co-existing domains for their frequency of occurrence in the human gene products (Additional file 7). As observed by the functions of co-existing domains, RRM containing gene products possess other RNA as well as protein binding domains, which might govern their specificity and affinity towards their RNA targets and assist them in performing their diverse biological functions.

### Enrichment analysis

**Figure 8:**
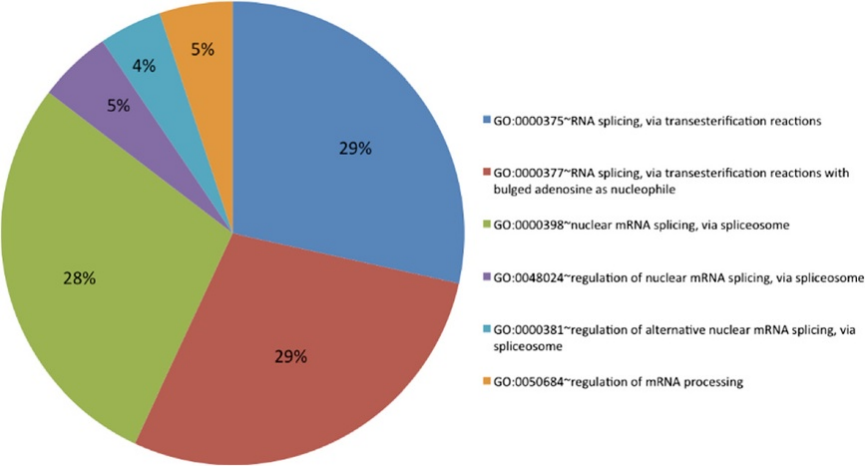
**Enrichment analysis for biological processes.** The RRM-containing human gene products were studied for their functional clustering based enrichment analysis using DAVID 6.7. The processes, which were observed to be enriched were related to mRNA processing, splicing and its regulation.

We studied the RRM domain-containing human gene products for their involvement in biological processes using DAVID 6.7 [35, 36]. They were observed to be involved in various processes involved in RNA metabolism. We further identified the biological processes, which were enriched in the RRM-containing gene products based on normalization using the biological processes performed by all the human gene products as background. Out of the set of 403 gene products, 173 are annotated with GO biological processes in DAVID 6.7. Upon functional clustering, these belong to 22 clusters. Upon filtering the results based on Bonferroni correction method (p < 0.05), 42 gene products were observed to belong to six biological processes performing mRNA processing and RNA splicing (Figure 8, Additional file 8).

Using DAVID 6.7, we also studied the KEGG pathway [37] enrichment in this set of human RRM-containing gene products. Upon performing functional clustering and using the same filtering parameters as explained above, 33 gene products were observed to be part of the spliceosome machinery (Additional file 9).

### Disease involvement and disorder content

The set of RRM-containing human gene products were further analyzed for their role in diseases and disorder content. We obtained a comprehensive list of RNA-binding proteins which are linked to Mendelian diseases in human (as recorded in OMIM database) from a recent review [38]. There are 157 RNA binding Ensembl gene models that are implicated in Mendelian diseases [38]. We mapped these to the RRM-containing gene products identified in our analysis. 14 of these RNA-binding proteins linked with Mendelian diseases contain RRM domain (Additional file 10).

As ageing is reported as a risk factor for neurodegeneration and the role of RNA-binding proteins is implicated in neurodegeneration [39–41], we analyzed the disorder content of the RRM-containing gene products. Their disorder content of was analyzed using DISOPRED [42]. 16% of the gene products (Additional file 11) were high (>0.7) in their disorder content (% of disordered residues) and such gene products could be involved in processes such as ageing [39–41].

## Conclusions

RNA-binding proteins govern gene regulatory events at the post-transcriptional level. There are several well-characterized RNA-binding motifs present in the protein partner. Of these, RRM are the most abundant in higher vertebrates. In the present work, the genome-wide survey for the presence of RRM-containing gene products was performed in the human proteome, employing computational approaches starting from the known RRM-containing sequences present in the Pfam database.

The seven RRM families in Pfam are derived based on the HMM-HMM comparisons using a gathering threshold (GA threshold). GA thresholds are Pfam-bit scores and estimates of significance of hits. We studied these families for their features. We observed:

1. Taxonomic representation: The majority of the sequences belonging to these families are present in Eukaryota, with few bacterial RRM-containing proteins in the family RRM_1. RRM_2 and RRM Pfam families are not present in the class Mammalia and are present only in plants and fungi.
2. Sequence features: The members within the families are more similar as compared to other family members, as expected. However, some of the members of RRM_1, RRM_5 and RRM_6 families share high (>50%) sequence identity.
3. The conservation of amino acid residues was studied using ConSurf and mapped on the protein structures from each of the RRM families. The conserved residues were localized on similar structural regions.

We identified 928 gene products (403 gene products at 98% sequence identity), which contain RRM domain in the human genome upon performing the genome-wide scan using profile-based sequence search methods. As documented in existing literature, RRM is an abundant domain in eukaryotes [7, 15, 43, 44] and we also observed that 50% of the reported RNA-binding proteins (860 RNA-binding proteins, experimentally characterized by isolating mRNA interactome) in the human genome from a recent study [45] possess RRM domain. Their full-length sequences were analyzed for domain architectures in order to understand their functional roles. As RNA-binding proteins are known to mediate variety of different interactions and regulatory functions, analyzing the domain architectures of these full-length gene products will provide an insight into understanding of their evolution and biological functions. RRM_1 domain is present in majority of these human gene products (79%). 60% of the gene products were observed to possess multiple domains (either multiple RRM or non-RRM co-existing domains). RRM-containing proteins are known to possess modular nature (multiple repeats of RRM) [9, 34]. The length of the linker between the different RRM domains is known to govern specificity of RNA-binding, since a single RRM domain can bind from only four to eight nucleotides [9]. Therefore, modular nature of these proteins confer specificity to bind the target RNA as the number of nucleotides identified by single RRM domain is too small to define a unique target. The non-RRM co-existing domains were observed to be involved in functions like developmental signaling, apoptosis, transcriptional regulation, splicing and alternative splicing suggesting fundamental cellular roles of such genes.

The set of RRM-containing human gene products were mapped for their biological processes and pathways. The biological processes which were enriched in these gene products were related to mRNA splicing and its regulation. 33 of gene products were involved in the spiceosomal pathway. There are more than 100 gene products that are known to be part of the spiceosome. It is recently reported that more than half of the proteins in the spliceosome are intrinsically disordered (when proteins are considered in isolation) [46, 47]. These intrinsically disordered proteins are also implicated in age related neurodegenerative diseases [39–41]. One of the RRM-containing human gene product that encodes for FUS protein (fused in sarcoma, 546 amino acids long), is predicted to possess only 50 amino acids, which form a folded structure [39]. We calculated the fraction of disorder residues (low sequence complexity, rich in hydrophilic and aromatic residues) in the human RRM-containing gene products and observed that 16% of these are rich in disordered regions. It will be interesting to follow if these intrinsically disordered regions become structured upon binding to their RNA targets.

Also, recently RRM domain proteins have been implicated in several Mendelian diseases [38] and are observed to possess prion-forming ability [10, 48]. Therefore, we mapped these gene products to OMIM database and 14 of the human gene products were linked to Mendelian diseases. This study will help in characterization of RRM-containing gene products in the human genome and to provide early bioinformatics view of their functions.

## Methods

### Human proteome

The entire proteome of *Homo sapiens,* comprising of both reviewed and unreviewed entries, was downloaded from UniProt FTP website (http://www.uniprot.org/downloads). This set of human sequences was used to perform the genome-wide survey.

### RRM families

To perform searches in the human genome, we collated the known RRM sequences from the protein family database [17–20] (PFam). Pfam clusters sequences on the basis of their sequence similarities (HMM based) into seven different families. We studied these families for their sequence-based features like sequence identities and length distributions. The family alignments were employed to identify conserved sequence motifs using ConSurf [27] and these motifs were mapped on the structures of the RRM families. The sequences belonging to these families were also studied for their taxonomic distributions in various kingdoms and classes.

### Search protocol and its validation

The RRM-containing sequences belonging to the seven PFam families were used to perform searches in the human genome. Based on the phylogenetic tree analysis, these families were clustered into 10 new distinct clusters. The profiles of the new clusters were employed to perform searches in the human genome.

The multiple sequence alignment for all the members for each of the clusters was performed using MUSCLE 3.8 [24]. All the phylogenetic trees were visualized using FigTree 1.4.0 [49]. Subsequently, the human genome was searched using sensitive profile-based sequence search methods, RPS-BLAST [28, 29] and HMMScan [30].

1. We built PSI-BLAST profiles (position-specific substitution matrix) for each cluster, using the alignment of cluster members as an input against NR database at an Evalue = 10^-10^. A database of profiles of all the clusters was generated. The human gene products were searched against this database of profiles using RPS-BLAST at Evalue = 10^-3^.
2. For each of the cluster, we also generated HMM profiles using the alignment as an input. The entire human proteome was also searched against the HMM profiles of all clusters to identify putative RRM-containing gene products using HMMScan and an Evalue = 10^-2^.

The RRM-containing gene products identified in the human genome were further subjected to validation using GO annotations available for the human proteome [33]. We filtered them based on GO terms, RNA binding and nucleotide binding including their child terms.

### Analysis of RBP identified in human genome

These full-length gene products that contain sequence signature for at least one RRM domain were analyzed for their length distributions.

1. Domain architectures

The identified gene products were further filtered to remove isoforms, fragments and highly similar sequences, by clustering them using BLASTCLUST [31, 32] at 98% sequence similarity over an area covering 50% of the length. We then studied the domain architectures using HMMScan against a database of entire Pfam HMM profiles at 10^-5^. The domain architectures were observed for RRM-repeats and non-RRM co-existing domains were noted for their functions. The schematic for domain architectures was drawn using the software DOG 1.0 [50].

2. Biological processes and pathways

The identified RRM-containing human gene products were also mapped to their biological processes and the enrichment study for these processes was performed using DAVID 6.7 [35, 36]. Upon functional clustering, the results were filtered based on Bonferroni correction (p <0.05). We also studied these gene products for their pathway mapping in the KEGG database [37] using DAVID 6.7 [35, 36].

3. Disease implications and disorder content

The gene products were further mapped to OMIM [51] database using DAVID 6.7 [35, 36]. The disorder analysis was performed using DISOPRED [42]. All the residues were analyzed for their disorder and the disorder content (fraction of disordered residues) for these gene products was calculated.

## Electronic supplementary material

Additional file 1: **Is a table listing the taxonomic representation of RRM families.** (PDF 54 KB)

Additional file 2: **Is a figure, which highlights the percent sequence identity across different RRM families (In the Additional file 2, r1 stands for RRM_1, r2 for RRM_2, r3 for RRM_3, r4 for RRM_4, r5 for RRM_5, r6 for RRM_6 and r for RRM family.** R1_r2 implies percent identity distribution between the members of RRM_1 and RRM_2 families and likewise for other combinations). (TIFF 134 KB)

Additional file 3: **Is a figure that shows the co-clustering between members belonging to different RRM families (A. Neighbor joining tree-using ClustalW, B.** Maximum-likelihood tress using PhyML and C. Neighbor joining tree using MEGA 6 and employing alignment derived from MUSCLE 3.8). The color code followed is: RRM_1: Blue, RRM_2: Brown, RRM_3: Red, RRM_4: Pink, RRM_5: Yellow, RRM_6: Green and RRM: Cyan. (TIFF 4 MB)

Additional file 4: **Is a table listing the RRM-containing gene products identified in the human genome.** (PDF 118 KB)

Additional file 5: **Is a table listing the single domain RRM-containing human gene products and their molecular functions.** (PDF 84 KB)

Additional file 6: **Is a table listing the non-RRM co-existing domains with their functions present in the set of human RRM-containing gene products.** (PDF 83 KB)

Additional file 7: **Is a figure that shows the frequency of co-existing domains in the RRM-containing human gene products.** (TIFF 122 KB)

Additional file 8: **Is a table listing the enriched GO functions in RRM-containing human gene products.** (PDF 76 KB)

Additional file 9: **Is a figure that highlights the gene products involved in spilceosome pathway (In red the spliceosome components that contain the gene products we identified upon genome-wide survey are marked).** The figure displays all the components that are known to be part of spliceosome pathway (as in KEGG). The gene products, which were identified using our search strategy, are marked with red stars. (TIFF 399 KB)

Additional file 10: **Is a table listing the 14 human RRM-containing gene products with implications in Mendelian diseases.** (PDF 51 KB)

Additional file 11: **Is a table listing the predicted disorder content in the RRM-containing gene products identified in the human genome.** (PDF 182 KB)
