# Supplementary material for: Sequence search and analysis of gene products containing RNA recognition motifs in the human genome
Source: BMC Genomics. 2014 Dec 22;15(1):1159. doi: 10.1186/1471-2164-15-1159 (PMC4367854; doi:10.1186/1471-2164-15-1159)
Supplement: Supplementary file 1 — Additional file 1: Is a table listing the taxonomic representation of RRM families. (PDF 54 KB) [file 12864_2014_6891_MOESM1_ESM.pdf]

Additional file 1: Taxonomic representation of RRM. The RRM families defined in Pfam were analyzed for their taxonomic distribution. Pfam families RRM_2 and RRM have no representation in human genome and in the Mammalia class.

Pfam family ID	Pfam family name	Total number of sequences in Eukaryota	Number of sequences in class Mammalia	Taxonomic distribution (Kingdom)
PF00076	RRM_1	32653	7564	Eukaryota (Metazoa, Viridiplantae and Fungi) and Bacteria
PF04059	RRM_2	376	-	Viridiplantae and Fungi
PF08777	RRM_3	305	102	Metazoa and Viridiplantae
PF10598	RRM_4	345	31	Metazoa and Fungi
PF13893	RRM_5	4497	1347	Metazoa, Viridiplantae and Fungi
PF14259	RRM_6	6690	1673	Metazoa, Viridiplantae and Fungi
PF10378	RRM	Fungal specific	-	Fungi
