# Supplementary figures and images for: Sequence search and analysis of gene products containing RNA recognition motifs in the human genome
Source: BMC Genomics. 2014 Dec 22;15(1):1159. doi: 10.1186/1471-2164-15-1159 (PMC4367854; doi:10.1186/1471-2164-15-1159)

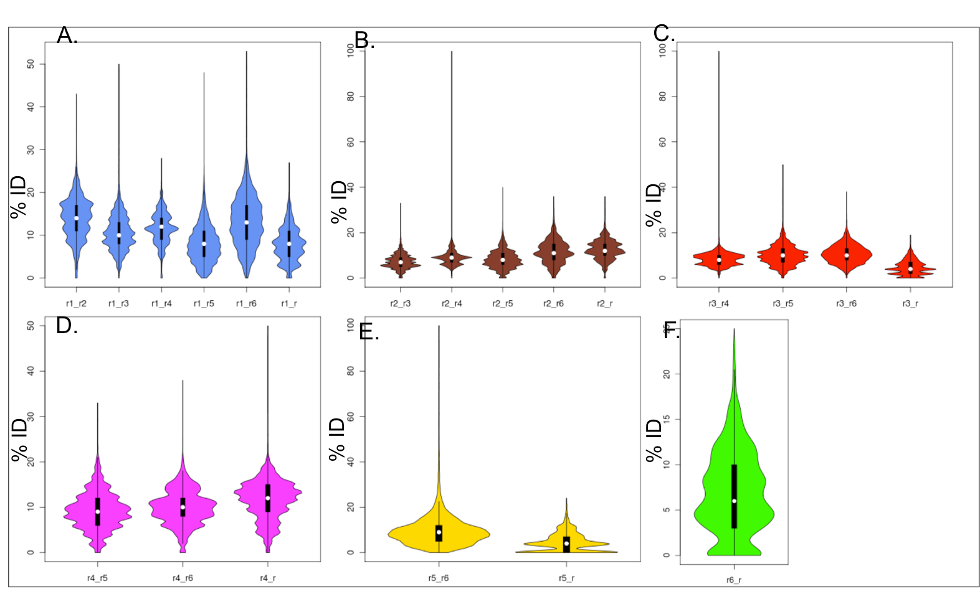

Supplement: Supplementary file 2 — Additional file 2: Is a figure, which highlights the percent sequence identity across different RRM families (In the Additional file 2, r1 stands for RRM_1, r2 for RRM_2, r3 for RRM_3, r4 for RRM_4, r5 for RRM_5, r6 for RRM_6 and r for RRM family. R1_r2 implies percent identity distribution between the members of RRM_1 and RRM_2 families and likewise for other combinations). (TIFF 134 KB) [file 12864_2014_6891_MOESM2_ESM.tiff]

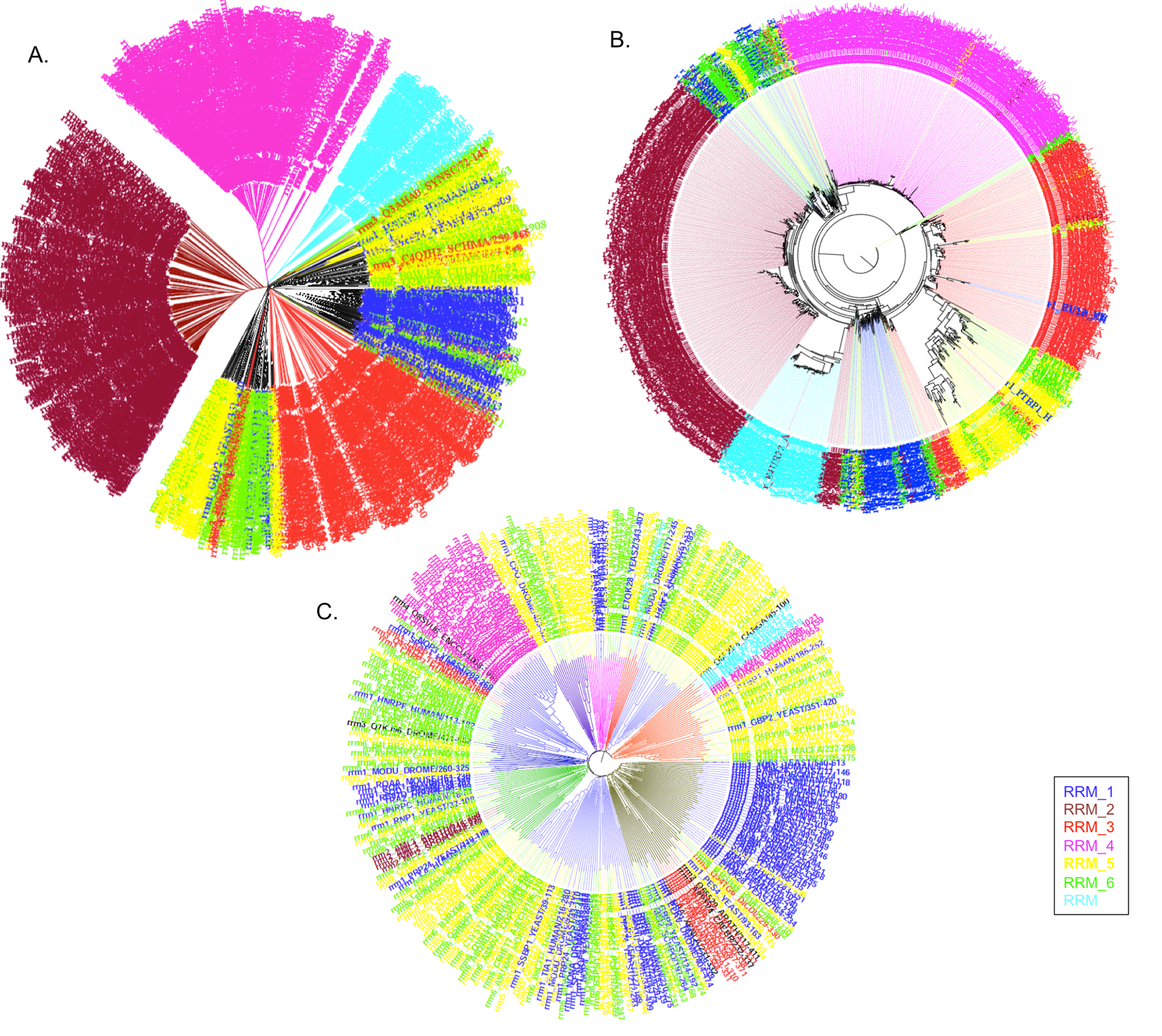

Supplement: Supplementary file 3 — Additional file 3: Is a figure that shows the co-clustering between members belonging to different RRM families (A. Neighbor joining tree-using ClustalW, B. Maximum-likelihood tress using PhyML and C. Neighbor joining tree using MEGA 6 and employing alignment derived from MUSCLE 3.8). The color code followed is: RRM_1: Blue, RRM_2: Brown, RRM_3: Red, RRM_4: Pink, RRM_5: Yellow, RRM_6: Green and RRM: Cyan. (TIFF 4 MB) [file 12864_2014_6891_MOESM3_ESM.tiff]

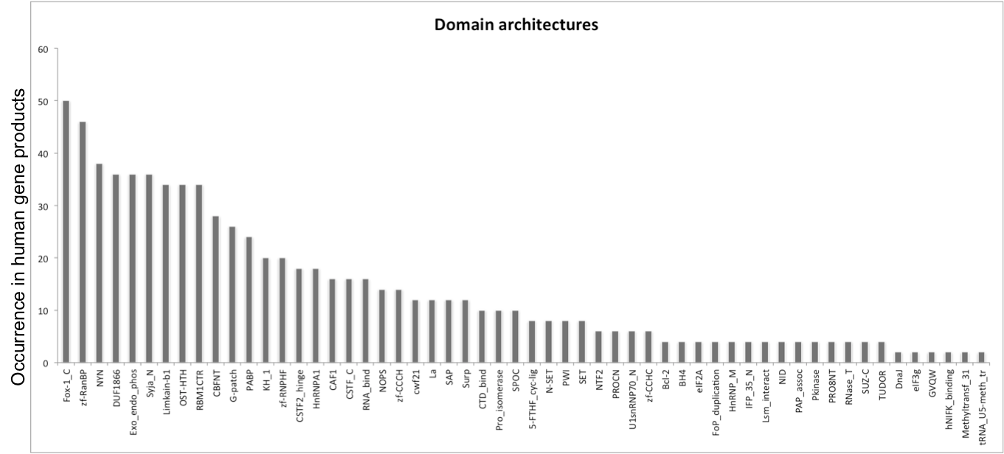

Supplement: Supplementary file 7 — Additional file 7: Is a figure that shows the frequency of co-existing domains in the RRM-containing human gene products. (TIFF 122 KB) [file 12864_2014_6891_MOESM7_ESM.tiff]

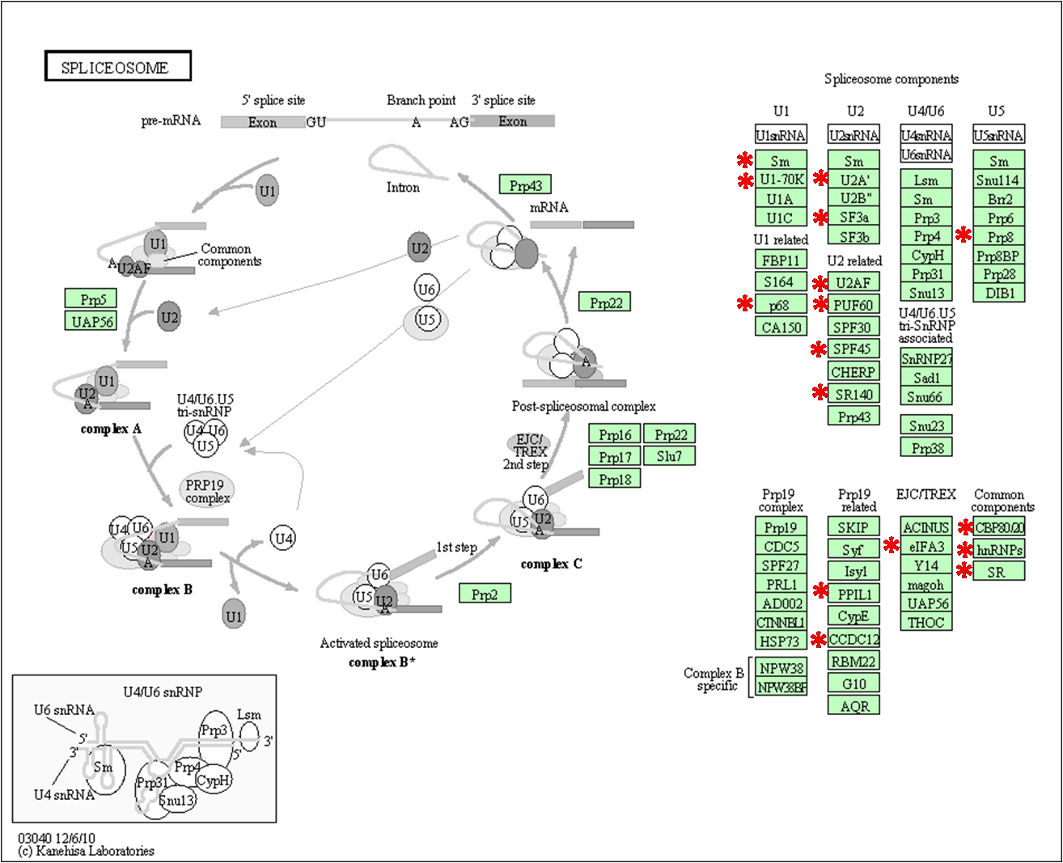

Supplement: Supplementary file 9 — Additional file 9: Is a figure that highlights the gene products involved in spilceosome pathway (In red the spliceosome components that contain the gene products we identified upon genome-wide survey are marked). The figure displays all the components that are known to be part of spliceosome pathway (as in KEGG). The gene products, which were identified using our search strategy, are marked with red stars. (TIFF 399 KB) [file 12864_2014_6891_MOESM9_ESM.tiff]
