# Supplementary material for: Sequence search and analysis of gene products containing RNA recognition motifs in the human genome
Source: BMC Genomics. 2014 Dec 22;15(1):1159. doi: 10.1186/1471-2164-15-1159 (PMC4367854; doi:10.1186/1471-2164-15-1159)
Supplement: Supplementary file 4 — Additional file 4: Is a table listing the RRM-containing gene products identified in the human genome. (PDF 118 KB) [file 12864_2014_6891_MOESM4_ESM.pdf]

Additional file 4: The *Homo sapiens* proteins (UniProt IDs) identified as RRM-containing using sequence searches.

A0AV96	E5RI26	G3V2S9	O60506-5	Q13310-3	Q8WUA2
A0AV96-2	E5RIX9	G3V3K6	O60812	Q13595	Q8WVV9
A2A2V2	E5RJ83	G3V4C1	O75494	Q13595-2	Q8WVV9-2
A2ABK1	E5RJB9	G3V4M8	O75494-2	Q14011	Q8WVV9-3
A6NDE4	E5RJM0	G3V4T2	O75494-3	Q14103	Q8WVV9-4
A6NDY0	E5RJM8	G3V4W0	O75494-4	Q14103-2	Q8WXA9
A6NDY0-2	E5RJV8	G3V4X6	O75494-5	Q14103-3	Q8WXA9-2
A6NDY0-4	E5RJW8	G3V546	O75526	Q14103-4	Q8WXF0
A6NEQ0	E7EN40	G3V555	O75821	Q14151	Q8WXF1
A6NFN3	E7EN82	G3V575	O95104	Q14498	Q8WXF1-2
A6NKZ9	E7ENA5	G3V576	O95104-2	Q14498-2	Q92804
A6NLN1	E7ENA6	G3V5K8	O95319	Q14498-3	Q92804-2
A6PVI3	E7EPF2	G3V5X6	O95319-2	Q14576	Q92843-2
A8K1C9	E7EPM3	G3V5Z6	O95319-3	Q14576-2	Q92879
A8MXP9	E7EQJ0	G3XAC6	O95319-4	Q14966	Q92879-2
B0LM41	E7EQS3	G3XAP0	O95319-5	Q14966-2	Q92879-3
B0QYK0	E7EQV3	G5E9J9	O95453	Q14966-3	Q92879-4
B0QYK1	E7ERJ7	G5E9M3	O95453-2	Q14966-4	Q92904
B0QYV1	E7ERQ6	G5EA30	O95453-3	Q14966-5	Q92904-2
B0QYY4	E7ET15	G8JLB6	O95628	Q15020	Q93062
B0QYY7	E7ET38	G8JLI7	O95628-2	Q15056	Q93062-2
B1AKF7	E7ETC0	G8JLP4	O95628-3	Q15056-2	Q93062-3
B1AKP7	E7ETJ9	H0Y3K3	O95628-4	Q15233	Q93062-4
B1ALY6	E7ETM7	H0Y4X3	O95628-5	Q15287	Q93062-5
B1AM48	E7ETR3	H0Y5F5	O95628-6	Q15287-2	Q96B58
B1AM49	E7ETU5	H0Y5U7	O95628-7	Q15287-3	Q96DH6
B1ANR0	E7EU30	H0Y623	O95628-8	Q15415	Q96DH6-2
B1ANR1	E7EU33	H0Y6E7	O95758	Q15415-2	Q96DH6-3
B1APY8	E7EU39	H0Y713	O95758-1	Q15424	Q96DU9
B1APY9	E7EU98	H0Y8D9	O95758-2	Q15427	Q96E39
B3KM87	E7EUF4	H0Y8G5	O95758-4	Q15434	Q96EP5
B3KSB0	E7EV40	H0Y8R1	O95758-5	Q15695	Q96EP5-2
B3KT61	E7EVG6	H0Y8T4	O95758-6	Q15696	Q96H35
B3KWE6	E7EW00	H0Y9D9	P05455	Q15717	Q96I25
B3KWU8	E7EWI9	H0YA61	P07910	Q16206	Q96IC2
B4DEH8	E7EWR4	H0YA82	P07910-2	Q16206-2	Q96IC2-2
B4DF29	E7EX17	H0YA96	P07910-4	Q16560	Q96IZ5
B4DFI3	E9PAM1	H0YAK1	P08579	Q16560-2	Q96IZ5-2
B4DHE8	E9PAU2	H0YAM1	P08621	Q16629	Q96J87

B4DHY1	E9PB47	H0YAP2	P08621-2	Q16629-2	Q96J87-2
B4DI28	E9PB51	H0YAQ2	P08621-3	Q16629-3	Q96J87-3
B4DIB6	E9PB61	H0YAR2	P09012	Q16630	Q96J87-4
B4DJ45	E9PBY2	H0YB39	P09651	Q16630-2	Q96LT9
B4DJK0	E9PC62	H0YB86	P09651-2	Q16630-3	Q96PK6
B4DM51	E9PCY7	H0YBB3	P09651-3	Q17RY0	Q96PK6-2
B4DMM2	E9PDD9	H0YBD7	P0C7P1	Q17RY0-2	Q96T37
B4DN88	E9PEG6	H0YBG7	P0CB38	Q17RY0-3	Q96T37-2
B4DN89	E9PEQ6	H0YBN4	P0DJD3	Q1RMF9	Q96T37-3
B4DQI6	E9PFH8	H0YBQ4	P0DJD3-2	Q2L7G6	Q96T58
B4DQL3	E9PFS2	H0YBR2	P0DJD4	Q2M296	Q99729
B4DS13	E9PGM9	H0YBU9	P11940	Q2M296-2	Q99729-2
B4DSB0	E9PGX9	H0YCC8	P11940-2	Q2PYN1	Q99729-3
B4DSS8	E9PIA5	H0YCP8	P14866	Q32P51	Q99729-4
B4DSU6	E9PID8	H0YEM1	P14866-2	Q49AS9	Q9BQ04
B4DTC1	E9PK21	H0YEQ8	P18615	Q4G0J3	Q9BRL6
B4DTC3	E9PKA1	H0YEU6	P19338	Q4G0J3-2	Q9BRL6-2
B4DUA4	E9PKU1	H0YEY9	P22626	Q4VXU2	Q9BRS8
B4DUA9	E9PL19	H0YGN7	P22626-2	Q4VY17	Q9BTD8
B4DUN1	E9PLB0	H0YH80	P23246	Q5H918	Q9BTD8-2
B4DVB8	E9PM61	H0YHB7	P23246-2	Q5H919	Q9BTD8-3
B4DWT1	E9PMU7	H0YHU8	P23588	Q5JB52	Q9BTD8-4
B4DY08	E9PN18	H0YIB4	P26368	Q5JQF3	Q9BWF3
B4DYX9	E9PQ56	H0YIL2	P26368-2	Q5JQF8	Q9BWF3-2
B4DZ27	E9PQK4	H0YJ07	P26378	Q5JRI1	Q9BWF3-3
B4E241	E9PQU5	H0YJ40	P26378-2	Q5JRI3	Q9BX46
B4E2X2	E9PSF1	H0YJH9	P26378-3	Q5QP21	Q9BY77
B4E3T4	E9PSH0	H0YJJ3	P26378-4	Q5QP22	Q9BY77-2
B7WPG3	F2Z2G3	H0YJU7	P26378-5	Q5QP23	Q9BYG3
B7Z1U7	F2Z2U1	H0YJW7	P26599	Q5QPL9	Q9BZB8
B7Z2F6	F2Z2W2	H0YKS1	P26599-2	Q5QPM1	Q9BZB8-2
B7Z2K5	F2Z2W7	H0YN19	P26599-3	Q5QPM2	Q9BZB8-3
B7Z888	F5GWK3	H3BMM9	P29558	Q5RL73	Q9BZB8-4
B7Z8Z7	F5GWN9	H3BMS0	P29558-2	Q5SZ64	Q9BZC1
B7ZKM0	F5GXV1	H3BNC1	P31483	Q5SZQ7	Q9BZC1-2
B7ZLQ8	F5GXV8	H3BNC9	P31483-2	Q5SZQ8	Q9BZC1-3
B7ZMD9	F5GY08	H3BNY3	P31942	Q5SZQ8-2	Q9BZC1-4
B8ZZ74	F5GYA8	H3BPE7	P31942-2	Q5SZQ8-3	Q9BZC1-5
B9ZVT1	F5GYZ3	H3BPG5	P31942-3	Q5T0W7	Q9GZT3
C9IYN3	F5GZT4	H3BPI5	P31942-4	Q5T481	Q9H0L4
C9IZL7	F5GZU3	H3BR27	P31942-5	Q5T8P6	Q9H0Z9
C9J286	F5H047	H3BR57	P31942-6	Q5T8P6-2	Q9H0Z9-2

C9J2Z9	F5H0D8	H3BRB1	P31943	Q5T8P6-3	Q9H361
C9J323	F5H0H3	H3BT71	P33240	Q5T8P6-4	Q9H6E5
C9J4X2	F5H0I5	H3BTC0	P33240-2	Q5T8P6-5	Q9H6T0
C9J6C5	F5H0M7	H3BUA9	P35637	Q5TGA3	Q9H6T0-2
C9J787	F5H0R1	H3BV80	P35637-2	Q5VV67	Q9HCJ3
C9J9B2	F5H101	H7BXE3	P38159	Q5VV67-2	Q9HCJ3-2
C9JAA9	F5H160	H7BXF3	P38159-2	Q5VZ19	Q9NQ94
C9JAB2	F5H330	H7BXH8	P38159-3	Q5VZ19-2	Q9NQ94-2
C9JB16	F5H357	H7BY16	P42696	Q5VZZ6	Q9NQ94-3
C9JBI6	F5H3W3	H7BY36	P42696-2	Q6NXG1	Q9NQ94-4
C9JE21	F5H4D6	H7BY49	P43243	Q6NXG1-2	Q9NQ94-5
C9JFZ1	F5H4Y5	H7BZE0	P43243-2	Q6NXG1-3	Q9NQ94-6
C9JGE3	F5H532	H7C367	P49756	Q6NXG1-4	Q9NQZ3
C9JIJ9	F5H540	H7C3F4	P49756-2	Q6P2Q9	Q9NR90
C9JJZ7	F5H5I6	H7C476	P49756-3	Q6XE24	Q9NR90-2
C9JKX0	F5H606	H7C5G8	P51991	Q6XE24-2	Q9NTZ6
C9JLZ0	F5H659	I3L0B6	P51991-2	Q6XE24-3	Q9NVM6
C9JM38	F5H669	I3L0J9	P52272	Q6XE24-4	Q9NW13
C9JQX9	F5H674	I3L1D4	P52272-2	Q6ZP01	Q9NW64
C9JT33	F5H6M0	I3L521	P52298	Q6ZRY4	Q9NW64-2
C9JTN7	F5H6R6	J3KMY7	P52298-2	Q76FK4	Q9NWB1
C9JYS8	F5H8B8	J3KN18	P52597	Q76FK4-4	Q9NWB1-2
C9JZG1	F6S8J2	J3KN28	P52756	Q7Z5Q1	Q9NWB1-3
C9K041	F6T1J1	J3KN81	P55795	Q7Z5Q1-3	Q9NWB1-4
D6R9D6	F6UBL3	J3KNW3	P55884	Q7Z5Q1-4	Q9NWB1-5
D6R9K7	F6VZ39	J3KP15	P55884-2	Q7Z5Q1-5	Q9NWH9
D6R9M7	F6W1U9	J3KPD3	P57052	Q7Z5Q1-6	Q9NX07
D6R9P3	F6WRY4	J3KPK1	P62995	Q7Z5Q1-7	Q9NX07-2
D6R9T0	F8VP89	J3KPK3	P62995-3	Q86SE5	Q9NZI8
D6R9Z6	F8VQP3	J3KQ82	P78332	Q86SG3	Q9P1R7
D6RA49	F8VR27	J3KQA8	P84103	Q86SG3-2	Q9P2K5
D6RAF8	F8VRQ1	J3KQV8	P98175	Q86U06	Q9P2K5-2
D6RAM1	F8VRS4	J3KSR8	P98175-2	Q86U06-2	Q9P2K5-3
D6RBI2	F8VRU1	J3KSW7	P98175-3	Q86U06-3	Q9P2N5
D6RBK5	F8VSB5	J3KTC1	P98175-4	Q86U06-4	Q9UBK2
D6RBM0	F8VSC7	J3KTH5	P98179	Q86U06-5	Q9UHX1
D6RBP6	F8VTQ5	J3KTL2	Q01081	Q86U42	Q9UHX1-2
D6RBQ9	F8VV01	J3QKT5	Q01081-2	Q86U42-2	Q9UHX1-3
D6RBS9	F8VV04	J3QKU3	Q01081-4	Q86V81	Q9UHX1-4
D6RBZ0	F8VVB8	J3QL05	Q01085	Q86YN6	Q9UHX1-5
D6RD18	F8VX11	J3QQV5	Q01085-2	Q86YN6-2	Q9UHX1-6
D6RD83	F8VXY0	J3QQZ2	Q01130	Q86YN6-3	Q9UKA9

D6RDL0	F8VXY6	J3QRF4	Q01844	Q86YN6-4	Q9UKA9-2
D6RDU3	F8VYE9	J3QRR5	Q01844-2	Q86YN6-5	Q9UKA9-3
D6RE02	F8VYN5	J3QT21	Q01844-3	Q86YN6-6	Q9UKA9-4
D6REM6	F8VZ49	J3QT54	Q01844-5	Q8IUH3	Q9UKA9-5
D6REZ6	F8VZG9	O00425	Q02040-2	Q8IUH3-2	Q9UKA9-6
D6RF41	F8VZY7	O14979	Q02040-3	Q8IUH3-3	Q9UKM9
D6RF44	F8W0K0	O14979-2	Q07955	Q8IXT5	Q9UKM9-2
D6RFF0	F8W1S7	O14979-3	Q07955-2	Q8IY67	Q9ULW3
D6RFL5	F8W1T6	O15042	Q07955-3	Q8IY67-2	Q9UN86
D6RFM3	F8W646	O15042-2	Q08170	Q8IYX4	Q9UN86-2
D6RGD8	F8W6I7	O15047	Q12849	Q8IZ69	Q9UNP9
D6RIA2	F8W8I6	O43251	Q12849-5	Q8IZ69-2	Q9UNP9-2
D6RIH9	F8W930	O43251-10	Q12926	Q8N684	Q9UPN6
D6RIT2	F8W940	O43251-2	Q12926-2	Q8N684-2	Q9UPS6
D6RIU0	F8W9F8	O43251-3	Q13117	Q8N684-3	Q9Y388
D6RJ04	F8WA97	O43251-4	Q13117-2	Q8N6W0	Q9Y3B4
D6W592	F8WB35	O43251-5	Q13117-3	Q8N6W0-2	Q9Y4C8
E1P5S2	F8WCA5	O43251-6	Q13148	Q8N7X1	Q9Y4F3
E2PSN0	F8WD15	O43251-7	Q13148-2	Q8N9W6	Q9Y4F3-3
E5RFD8	F8WD91	O43251-8	Q13148-3	Q8N9W6-2	Q9Y4F3-4
E5RFP2	F8WDX3	O43251-9	Q13151	Q8N9W6-3	Q9Y4F3-5
E5RFV3	F8WE16	O43347	Q13242	Q8N9W6-4	Q9Y580
E5RG67	F8WE42	O43390	Q13243	Q8NDT2	Q9Y5S9
E5RG71	F8WJN3	O43390-2	Q13243-2	Q8NE35	Q9Y5S9-2
E5RGC4	G3V129	O43426	Q13243-3	Q8NE35-2	Q9Y6M1
E5RGH3	G3V162	O43426-2	Q13247	Q8TAS1	Q9Y6M1-1
E5RGH4	G3V1M1	O43426-4	Q13247-2	Q8TBF4	Q9Y6M1-3
E5RGV0	G3V1V1	O43719	Q13247-3	Q8TBY0	Q9Y6M1-4
E5RGV5	G3V251	O60506	Q13283	Q8TC92	Q9Y6M1-5
E5RH24	G3V2B4	O60506-2	Q13287	Q8WU68	Q9Y6M1-6
E5RHG1	G3V2H6	O60506-3	Q13310	Q8WU68-2	
E5RHG7	G3V2Q1	O60506-4	Q13310-2	Q8WU68-3	
