# Supplementary material for: Sequence search and analysis of gene products containing RNA recognition motifs in the human genome
Source: BMC Genomics. 2014 Dec 22;15(1):1159. doi: 10.1186/1471-2164-15-1159 (PMC4367854; doi:10.1186/1471-2164-15-1159)
Supplement: Supplementary file 5 — Additional file 5: Is a table listing the single domain RRM-containing human gene products and their molecular functions. (PDF 84 KB) [file 12864_2014_6891_MOESM5_ESM.pdf]

Additional file 5: GO biological process of single domain containing proteins. The single domain containing proteins were analyzed for their GO biological processes (BP). 153 proteins possess only one domain and only 39 of these have annotated GO BP. These map to 145 unique biological processes.

**Single RRM containing proteins**	**Uniprot ID**	**GO biological process**	**Pfam domain**
Nuclear cap-binding protein subunit 2-like	A6PVI3	GO:0045292	RRM_1
Polyadenylate-binding protein 1	E5RJB9	GO:0031047<br>GO:0060213<br>GO:1900153	RRM_1
Lupus La protein	E9PGX9	GO:0006396	RRM_1
CUGBP Elav-like family member 1	E9PKU1	GO:0006376<br>GO:0007286<br>GO:0040018	RRM_1
Squamous cell carcinoma antigen recognized by T-cells 3	F8VV04	GO:0006396	RRM_1
Nucleolysin TIA-1 isoform p40	F8WE16	GO:0017148<br>GO:0042036	RRM_1
RNA-binding protein 39	H0Y4X3	GO:0006397	RRM_1
Serine/arginine-rich splicing factor 10	O75494	GO:0000244<br>GO:0000375<br>GO:0000398<br>GO:0006355<br>GO:0006376<br>GO:0006406<br>GO:0016482<br>GO:0048025	RRM_1
Heterogeneous nuclear ribonucleoproteins C1/C2	P07910	GO:0000398<br>GO:0008380<br>GO:0010467	RRM_1

Nuclear cap-binding protein subunit 2	P52298	GO:0000184<br>GO:0000387<br>GO:0000398<br>GO:0006366<br>GO:0006368<br>GO:0006369<br>GO:0006370<br>GO:0006406<br>GO:0006408<br>GO:0006446<br>GO:0008334<br>GO:0008380<br>GO:0010467<br>GO:0016032<br>GO:0016070<br>GO:0016071<br>GO:0031047<br>GO:0031124<br>GO:0034660<br>GO:0045292<br>GO:0046833<br>GO:0050434	RRM_1
Splicing regulator RBM11	P57052	GO:0000381<br>GO:0006397<br>GO:0007275<br>GO:0008380<br>GO:0030154<br>GO:0034599	RRM_1
Transformer-2 protein homolog beta	P62995	GO:0000375<br>GO:0000381<br>GO:0000398<br>GO:0048026	RRM_1
Serine/arginine-rich splicing factor 3	P84103	GO:0000398<br>GO:0006366<br>GO:0006369<br>GO:0006406<br>GO:0008380<br>GO:0010467<br>GO:0031124	RRM_1
Putative RNA-binding protein 3	P98179	GO:0006396<br>GO:0006412<br>GO:0006417<br>GO:0009409<br>GO:0035196<br>GO:0045727	RRM_1

Serine/arginine-rich splicing factor 2	Q01130	GO:0000398<br>GO:0006366<br>GO:0006369<br>GO:0006397<br>GO:0006406<br>GO:0008380<br>GO:0010467<br>GO:0031124<br>GO:2001141	RRM_1
Transformer-2 protein homolog alpha	Q13595	GO:0000398	RRM_1
Cold-inducible RNA-binding protein	Q14011	GO:0009409<br>GO:0009411<br>GO:0034063<br>GO:0045727<br>GO:0048255	RRM_1
Eukaryotic translation initiation factor 4H	Q15056	GO:0006412<br>GO:0006413<br>GO:0006446<br>GO:0010467<br>GO:0016032<br>GO:0019953<br>GO:0044267<br>GO:0048589	RRM_1
RNA-binding protein with serine-rich domain 1	Q15287	GO:0000184<br>GO:0000381<br>GO:0000398<br>GO:0006351<br>GO:0006366<br>GO:0006369<br>GO:0006406<br>GO:0008380<br>GO:0010467<br>GO:0016070<br>GO:0016071<br>GO:0031124<br>GO:0043065<br>GO:0048025	RRM_1
Ecto-NOX disulfide-thiol exchanger 2	Q16206	GO:0007624<br>GO:0016049<br>GO:0040008<br>GO:0055114	RRM_1

Serine/arginine-rich splicing factor 7	Q16629	GO:0000398 GO:0006366 GO:0006369 GO:0006397 GO:0006406 GO:0008380 GO:0010467 GO:0031124 GO:0048025	RRM_1
Peroxisome proliferator-activated receptor gamma coactivator-related protein 1	Q5VV67	GO:0006351 GO:0006355	RRM_1
Peroxisome proliferator-activated receptor gamma coactivator 1-beta	Q86YN6	GO:0001503 GO:0006355 GO:0006366 GO:0006390 GO:0007015 GO:0010469 GO:0010694 GO:0030520 GO:0034614 GO:0042327 GO:0044255 GO:0044281 GO:0045672 GO:0045780 GO:0045892 GO:0045944 GO:0051384 GO:0051591 GO:0060346 GO:2000273	RRM_1
Zinc finger CCHC-type and RNA-binding motif-containing protein 1	Q8TBF4	GO:0006397 GO:0008380	RRM_1
Ecto-NOX disulfide-thiol exchanger 1	Q8TC92	GO:0048511	RRM_1
Serine/arginine-rich splicing factor 12	Q8WXF0	GO:0000244 GO:0000381 GO:0000395 GO:0016482 GO:0048025	RRM_1
Serine/arginine-rich splicing factor 8	Q9BRL6	GO:0006397 GO:0008380	RRM_1

RNA-binding protein 24	Q9BX46	GO:0010830<br>GO:0030154<br>GO:0043488	RRM_1
Polymerase delta-interacting protein 3	Q9BY77	GO:0016973<br>GO:0045727	RRM_1
RNA-binding protein 38	Q9H0Z9	GO:0006397<br>GO:0006977<br>GO:0006978<br>GO:0007049<br>GO:0007050<br>GO:0008285<br>GO:0008380<br>GO:0010830<br>GO:0030154<br>GO:0043484<br>GO:0070935	RRM_1
Pre-mRNA-splicing factor RBM22	Q9NW64	GO:0000060<br>GO:0000398<br>GO:0033120<br>GO:0035690<br>GO:0045292<br>GO:0090316	RRM_1
Peroxisome proliferator-activated receptor gamma coactivator 1-alpha	Q9UBK2	GO:0001659<br>GO:0001678<br>GO:0006012<br>GO:0006094<br>GO:0006355<br>GO:0006367<br>GO:0006397<br>GO:0006461<br>GO:0007005<br>GO:0007586<br>GO:0008209<br>GO:0008380<br>GO:0009409<br>GO:0010822<br>GO:0010941<br>GO:0014850<br>GO:0019395<br>GO:0022904<br>GO:0030521<br>GO:0034599<br>GO:0035066<br>GO:0036273<br>GO:0042594<br>GO:0043201<br>GO:0043524<br>GO:0045333	RRM_1

		GO:0045722<br>GO:0045820<br>GO:0045893<br>GO:0045944<br>GO:0046321<br>GO:0048661<br>GO:0050821<br>GO:0050873<br>GO:0051091<br>GO:0051552<br>GO:0070997<br>GO:0071250<br>GO:0071356<br>GO:0071398<br>GO:0071456<br>GO:0071871<br>GO:0071873<br>GO:0097067<br>GO:1901857<br>GO:1901860<br>GO:1901863<br>GO:2000272<br>GO:2000310<br>GO:2000507<br>GO:2001171	
RNA-binding protein Raly	Q9UKM9	GO:0000398	RRM_1
Pre-mRNA branch site protein p14	Q9Y3B4	GO:0000398<br>GO:0008380<br>GO:0010467	RRM_1
RNA-binding protein 8A	Q9Y5S9	GO:0000184<br>GO:0000381<br>GO:0000398<br>GO:0006366<br>GO:0006369<br>GO:0006406<br>GO:0006417<br>GO:0008380<br>GO:0010467<br>GO:0016070<br>GO:0016071<br>GO:0031124	RRM_1
Nucleolar protein 8	Q76FK4	GO:0006260<br>GO:0006364<br>GO:0030307	RRM_6

Cleavage and polyadenylation specificity factor subunit 6	Q16630	GO:0006378<br>GO:0006397<br>GO:0051262	RRM_6
RNA-binding protein 27	Q9P2N5	GO:0006397	RRM_5
RNA-binding protein 20	Q5T481	GO:0006397<br>GO:0007507<br>GO:0008380<br>GO:0033120	RRM_5
