# Supplementary material for: Sequence search and analysis of gene products containing RNA recognition motifs in the human genome
Source: BMC Genomics. 2014 Dec 22;15(1):1159. doi: 10.1186/1471-2164-15-1159 (PMC4367854; doi:10.1186/1471-2164-15-1159)
Supplement: Supplementary file 6 — Additional file 6: Is a table listing the non-RRM co-existing domains with their functions present in the set of human RRM-containing gene products. (PDF 83 KB) [file 12864_2014_6891_MOESM6_ESM.pdf]

Additional file 6: Non-RRM co-existing domains. In some of the full-length RRM domain containing human proteins, non-RRM domains were also present. The table lists the 56 non-RRM domains, which co-exist with RRM domains and their associated functions.

**Domain**	**Function**
5-FTHF_cyc-lig	Biosynthesis of folic acid conatining compounds
Bcl-2	Apoptosis
BH4	Apoptosis
CAF1	Rnase; 3' to 5' mRNA deadenylation
CBFNT	Transcriptional regulation
CSTF_C	Transcription termination
CSTF2_hinge	Polyadenylation
CTD_bind	RNA polymerase II-binding domain
cwf21	mRNA splicing
DnaJ	Chaperone
DUF1866	Unknown function
eIF2A	Transaltion initiation
eIF3g	Transaltion initiation
Exo_endo_phos	Intracellular signaling; Endo and exonuclease
FoP_duplication	Endonuclease-reverse transcriptase
Fox-1_C	Regulation of alternative splicing
G-patch	Present in DNA-repair proteins
GVQW	Conserved but unknown function
hNIFK_binding	Cell cycle regulation
HnRNP_M	Regulation of splicing
HnRNPA1	Regulation of alternative splicing
IFP_35_N	Antiviral
KH_1	Diverse RNA binding function
La	Regulation of translation
Limkain-b1	Monitoring of autoimmune diseases
Lsm_interact	pre m-RNA splicing
Methyltransf_31	Methyltransferase
N-SET	Regulation of gene expression
NID	Antiviral
NOPS	Transcriptional regulation
NTF2	Nuclear transport
NYN	Components of the processome/degradosome that process tRNAs or ribosomal RNAs.
OST-HTH	Nucleate or organise structurally related ribonucleoprotein (RNP) complexes

PABP	Translation initiation and mRNA stabilisation/degradation
PAP_assoc	Polynucleotide adenylyltransferase
Pkinase	Protein kinases; diversity of functions including cellualr signaling
Pro_isomerase	Peptidyl-prolyl isomerisation, chaperone and cell signaling
PRO8NT	pre m-RNA splicing
PROCN	pre m-RNA splicing
PWI	pre-mRNA processing
RBM1CTR	m-RNA binding
RNA_bind	Unknown function
RNase_T	End-turnover of tRNA
SAP	Chromosomal organization
SET	Protein-protein interaction domain
SPOC	Develpmental signaling
Surp	Regulation of alternative splicing
SUZ-C	Centrosome size regulation
Syja_N	Regulation of secretory pathway
tRNA_U5-meth_tr	tRNA maturation
TUDOR	Develpmental signaling
U1snRNP70_N	Splicing
zf-CCCH	Cell cycle regulation
zf-CCHC	DNA packaging
zf-RanBP	Nuclear transport
zf-RNPHF	Unknown function
