# Supplementary material for: Sequence search and analysis of gene products containing RNA recognition motifs in the human genome
Source: BMC Genomics. 2014 Dec 22;15(1):1159. doi: 10.1186/1471-2164-15-1159 (PMC4367854; doi:10.1186/1471-2164-15-1159)
Supplement: Supplementary file 8 — Additional file 8: Is a table listing the enriched GO functions in RRM-containing human gene products. (PDF 76 KB) [file 12864_2014_6891_MOESM8_ESM.pdf]

Additional file 8: GO enrichment analysis.

The human proteins identified as RRM- containing were further analyzed to identify the most populated GO biological processes in which these proteins are involved. There were six enriched GO biological processes and 42 human proteins map to these processes.

Uniprot Accession	Gene Name
772535	U2 small nuclear RNA auxiliary factor 2
773774	small nuclear ribonucleoprotein polypeptide B"
776010	trinucleotide repeat containing 4
776781	transformer 2 alpha homolog (Drosophila)
777820	small nuclear ribonucleoprotein 70kDa (U1)
778766	splicing factor, arginine/serine-rich 7, 35kDa
781879	bruno-like 6, RNA-binding protein (Drosophila)
782425	heterogeneous nuclear ribonucleoprotein A0
782499	RNA-binding motif protein 5
784244	PRP8 pre-mRNA processing factor 8 homolog (S. cerevisiae)
784420	heterogeneous nuclear ribonucleoprotein C (C1/C2)
785199	ribosomal protein L36a pseudogene 51; ribosomal protein L36a pseudogene 37; ribosomal protein L36a pseudogene 49; heterogeneous nuclear ribonucleoprotein H2 (H'); ribosomal protein L36a
785327	RNA-binding motif protein 25
785995	cleavage stimulation factor, 3' pre-RNA, subunit 2, 64kDa
786739	U2 small nuclear RNA auxiliary factor 1
791398	heterogeneous nuclear ribonucleoprotein M
794028	RNA-binding motif protein 8A
794968	splicing factor, arginine/serine-rich 5
795511	splicing factor 3B, 14 kDa subunit
797048	transformer 2 beta homolog (Drosophila)
798354	small nuclear ribonucleoprotein polypeptide A
799658	heterogeneous nuclear ribonucleoprotein D (AU-rich element RNA-binding protein 1, 37kDa)
800702	similar to RNA-binding motif protein, X-linked; similar to hCG2011544; RNA-binding motif protein, X-linked
802909	heterogeneous nuclear ribonucleoprotein H3 (2H9)
806156	splicing factor, arginine/serine-rich 6; similar to arginine/serine-rich splicing factor 6
810069	heterogeneous nuclear ribonucleoprotein A3
810866	serine-arginine repressor protein (35 kDa)
811567	splicing factor, arginine/serine-rich 3

812172	similar to heterogeneous nuclear ribonucleoprotein L-like; heterogeneous nuclear ribonucleoprotein L
812300	similar to ribonucleic acid binding protein S1; RNA-binding protein S1, serine-rich domain
812576	splicing factor, arginine/serine-rich 2
813541	heterogeneous nuclear ribonucleoprotein F
814227	heterogeneous nuclear ribonucleoprotein A2/B1
815252	bruno-like 4, RNA-binding protein (Drosophila)
816065	splicing factor, arginine/serine-rich 9
816714	FUS interacting protein (serine/arginine-rich) 1; similar to FUS interacting protein (serine-arginine rich) 1
818979	splicing factor 3b, subunit 4, 49kDa
822152	RNA-binding motif protein 15B
824519	heterogeneous nuclear ribonucleoprotein A1-like 3; similar to heterogeneous nuclear ribonucleoprotein A1; heterogeneous nuclear ribonucleoprotein A1 pseudogene 2; heterogeneous nuclear ribonucleoprotein A1; heterogeneous nuclear ribonucleoprotein A1 pseudogene
825011	splicing factor, arginine/serine-rich 4
825717	nuclear cap binding protein subunit 2, 20kDa
825901	polypyrimidine tract binding protein 1
