# Supplementary material for: Sequence search and analysis of gene products containing RNA recognition motifs in the human genome
Source: BMC Genomics. 2014 Dec 22;15(1):1159. doi: 10.1186/1471-2164-15-1159 (PMC4367854; doi:10.1186/1471-2164-15-1159)
Supplement: Supplementary file 10 — Additional file 10: Is a table listing the 14 human RRM-containing gene products with implications in Mendelian diseases. (PDF 51 KB) [file 12864_2014_6891_MOESM10_ESM.pdf]

Additional file 10: RRM containing gene products involved in Mendelian diseases.

**Ensembl ID**	**OMIM name**
ENSG00000015479	Myopathy, Distal, 2; Mpd2
ENSG00000075856	Porokeratosis, Disseminated Superficial Actinic, 1; Dsap1
ENSG00000089280	Histiocytoma, Angiomatoid Fibrous; Amyotrophic Lateral Sclerosis 6, With Or Without Frontotemporal Dementia;
ENSG00000100836	Oculopharyngeal Muscular Dystrophy; Opmd
ENSG00000106344	Alopecia, Neurologic Defects, And Endocrinopathy Syndrome
ENSG00000120948	Amyotrophic Lateral Sclerosis 10, With Or Without Frontotemporal Dementia;
ENSG00000174231	Retinitis Pigmentosa 13; Rp13
ENSG00000182872	Tarp Syndrome; Tarps
ENSG00000182944	Ewing Sarcoma; Es; Histiocytoma, Angiomatoid Fibrous
ENSG00000187191	Spermatogenic Failure, Y-Linked, 2; Spgfy2
ENSG00000188120	Spermatogenic Failure, Y-Linked, 2; Spgfy2
ENSG00000203867	Cardiomyopathy, Dilated, 1dd; Cmd1dd
ENSG00000205916	Spermatogenic Failure, Y-Linked, 2; Spgfy2
ENSG00000205944	Spermatogenic Failure, Y-Linked, 2; Spgfy2
