# Supplementary material for: Sequence search and analysis of gene products containing RNA recognition motifs in the human genome
Source: BMC Genomics. 2014 Dec 22;15(1):1159. doi: 10.1186/1471-2164-15-1159 (PMC4367854; doi:10.1186/1471-2164-15-1159)
Supplement: Supplementary file 11 — Additional file 11: Is a table listing the predicted disorder content in the RRM-containing gene products identified in the human genome. (PDF 182 KB) [file 12864_2014_6891_MOESM11_ESM.pdf]

Additional file 11: Disorder content in RRM containing proteins. We analyzed the human RRM domain containing proteins for their disorder content using DISOPRED.

Protein	Total residues	Disordered	Ordered	% Disorder	% Order
H7C3F4	600	553	47	0.92	0.08
Q9NWH9	1034	951	83	0.92	0.08
Q15424	915	806	109	0.88	0.12
Q14151	953	837	116	0.88	0.12
Q96T58	3664	3214	450	0.88	0.12
B4DS13	572	495	77	0.87	0.13
A6NDE4	496	425	71	0.86	0.14
Q92804	592	507	85	0.86	0.14
Q01844-5	661	559	102	0.85	0.15
B7ZMD9	459	387	72	0.84	0.16
P38159-2	378	318	60	0.84	0.16
B0QYK0	618	518	100	0.84	0.16
Q14966	1978	1656	322	0.84	0.16
B0QYK1	600	500	100	0.83	0.17
C9JGE3	583	483	100	0.83	0.17
Q5VV67	1664	1373	291	0.83	0.17
E7EX17	616	501	115	0.81	0.19
O75526	392	318	74	0.81	0.19
P38159	391	317	74	0.81	0.19
Q96E39	390	316	74	0.81	0.19
H3BPE7	527	425	102	0.81	0.19
P49756	843	672	171	0.80	0.20
Q9P2N5	1060	840	220	0.79	0.21
Q5T481	1227	964	263	0.79	0.21
O95104	1147	900	247	0.78	0.22
O95104-2	1125	878	247	0.78	0.22
Q76FK4	1167	906	261	0.78	0.22
Q86YN6	1023	790	233	0.77	0.23
Q5T8P6	1007	776	231	0.77	0.23
Q9UPS6	1923	1479	444	0.77	0.23
Q5T8P6-2	983	753	230	0.77	0.23
H7C5G8	186	142	44	0.76	0.24
Q9H0L4	616	469	147	0.76	0.24
Q15287	305	232	73	0.76	0.24
Q96PK6	669	508	161	0.76	0.24
Q08170	494	375	119	0.76	0.24
O15047	1707	1285	422	0.75	0.25
P62995	288	216	72	0.75	0.25
E7EWR4	597	446	151	0.75	0.25
P33240	577	431	146	0.75	0.25
H7BXE3	453	338	115	0.75	0.25
H0Y8D9	439	327	112	0.74	0.26

Q13595	282	210	72	0.74	0.26
J3KPD3	267	198	69	0.74	0.26
Q76FK4-4	1129	834	295	0.74	0.26
B7Z888	1337	987	350	0.74	0.26
P33240-2	560	413	147	0.74	0.26
Q9BRL6	282	207	75	0.73	0.27
P57052	281	206	75	0.73	0.27
P07910	306	223	83	0.73	0.27
Q9BY77	421	305	116	0.72	0.28
Q15287-3	268	194	74	0.72	0.28
Q9BRL6-2	275	199	76	0.72	0.28
Q9BYG3	293	212	81	0.72	0.28
F8VX11	360	260	100	0.72	0.28
Q9UKM9	306	221	85	0.72	0.28
Q16630	551	396	155	0.72	0.28
Q16630-2	588	421	167	0.72	0.28
Q9BY77-2	392	280	112	0.71	0.29
Q16629	238	170	68	0.71	0.29
A8MXP9	895	639	256	0.71	0.29
Q8WXF0	261	186	75	0.71	0.29
B4DYX9	387	275	112	0.71	0.29
Q15056	248	175	73	0.71	0.29
Q9UBK2	798	563	235	0.71	0.29
O95628-6	767	539	228	0.70	0.30
O75494	262	184	78	0.70	0.30
O15042	1029	722	307	0.70	0.30
H0YA82	334	234	100	0.70	0.30
P43243	847	593	254	0.70	0.30
Q9UKM9-2	290	202	88	0.70	0.30
H3BMS0	257	179	78	0.70	0.30
P98175	930	644	286	0.69	0.31
O43719	755	520	235	0.69	0.31
A2A2V2	408	281	127	0.69	0.31
P23246	707	486	221	0.69	0.31
E7EPM3	1007	689	318	0.68	0.32
Q9Y388	322	220	102	0.68	0.32
O60812	293	200	93	0.68	0.32
P07910-2	293	200	93	0.68	0.32
Q15696	482	328	154	0.68	0.32
Q9ULW3	272	185	87	0.68	0.32
B4DUN1	350	238	112	0.68	0.32
P98175-3	853	580	273	0.68	0.32
E9PB61	264	179	85	0.68	0.32
F5H160	1034	701	333	0.68	0.32
P52272	730	493	237	0.68	0.32
Q17RY0	729	492	237	0.67	0.33

Q8N684-3	514	346	168	0.67	0.33
Q15695	479	321	158	0.67	0.33
Q16206	610	407	203	0.67	0.33
B0LM41	339	226	113	0.67	0.33
Q15056-2	228	152	76	0.67	0.33
H0YN19	290	193	97	0.67	0.33
Q8TC92	643	427	216	0.66	0.34
J3QT21	434	288	146	0.66	0.34
P52756	815	540	275	0.66	0.34
B3KT61	280	185	95	0.66	0.34
Q8TBF4	217	143	74	0.66	0.34
H3BV80	211	139	72	0.66	0.34
H0YEU6	249	164	85	0.66	0.34
Q96IZ5	413	272	141	0.66	0.34
Q17RY0-2	712	468	244	0.66	0.34
Q13247	344	226	118	0.66	0.34
Q01130	221	145	76	0.66	0.34
G3V129	304	199	105	0.65	0.35
Q96EP5	407	266	141	0.65	0.35
D6RD83	92	60	32	0.65	0.35
H7BY36	308	200	108	0.65	0.35
Q8NE35	698	453	245	0.65	0.35
Q15427	424	275	149	0.65	0.35
H0YB86	165	107	58	0.65	0.35
J3QRF4	358	232	126	0.65	0.35
P08621	437	283	154	0.65	0.35
B4E3T4	224	145	79	0.65	0.35
P08621-2	428	276	152	0.64	0.36
Q9HCJ3	691	445	246	0.64	0.36
Q9BWF3	364	234	130	0.64	0.36
O43251-8	451	289	162	0.64	0.36
Q96J87	481	308	173	0.64	0.36
A6NDY0	278	178	100	0.64	0.36
P42696	430	275	155	0.64	0.36
Q9BQ04	359	228	131	0.64	0.36
Q96I25	401	254	147	0.63	0.37
B4DN89	209	132	77	0.63	0.37
Q7Z5Q1-3	575	360	215	0.63	0.37
H0YBD7	195	122	73	0.63	0.37
B4E2X2	263	163	100	0.62	0.38
A6NDY0-4	289	179	110	0.62	0.38
F8VP89	275	170	105	0.62	0.38
Q8WXA9-2	624	385	239	0.62	0.38
Q9P2K5	600	370	230	0.62	0.38
Q6XE24	437	269	168	0.62	0.38
Q8NE35-2	684	421	263	0.62	0.38

Q8IXT5	1001	616	385	0.62	0.38
Q86U06	439	270	169	0.62	0.38
H7BXF3	127	78	49	0.61	0.39
P49756-3	300	184	116	0.61	0.39
Q9P2K5-3	188	115	73	0.61	0.39
P19338	710	433	277	0.61	0.39
Q7Z5Q1-7	548	334	214	0.61	0.39
Q96J87-4	344	209	135	0.61	0.39
Q8IY67	606	368	238	0.61	0.39
E9PAU2	756	459	297	0.61	0.39
Q8N9W6-3	295	179	116	0.61	0.39
Q96H35	190	115	75	0.61	0.39
Q9BRS8	491	297	194	0.60	0.40
C9J4X2	124	75	49	0.60	0.40
P51991	378	228	150	0.60	0.40
Q92904-2	315	190	125	0.60	0.40
O43347	362	218	144	0.60	0.40
P22626	353	212	141	0.60	0.40
P09651	372	223	149	0.60	0.40
Q9BZC1	486	291	195	0.60	0.40
P84103	164	98	66	0.60	0.40
Q15233	471	281	190	0.60	0.40
B4DQL3	260	155	105	0.60	0.40
Q8WXF1	523	311	212	0.59	0.41
Q9BTD8-3	451	268	183	0.59	0.41
Q5QP21	202	120	82	0.59	0.41
H0YHB7	233	138	95	0.59	0.41
A6NDY0-2	255	151	104	0.59	0.41
Q9P2K5-2	576	341	235	0.59	0.41
Q9NW64	420	248	172	0.59	0.41
O95319-3	521	307	214	0.59	0.41
Q9BTD8	480	282	198	0.59	0.41
Q8N6W0-2	409	240	169	0.59	0.41
Q96J87-3	454	264	190	0.58	0.42
Q96T37	977	568	409	0.58	0.42
Q9UN86	482	280	202	0.58	0.42
Q15434	407	236	171	0.58	0.42
B4DN88	419	242	177	0.58	0.42
E9PK21	208	120	88	0.58	0.42
F5GWK3	371	214	157	0.58	0.42
Q9NWB1-3	370	213	157	0.58	0.42
Q14011	172	99	73	0.58	0.42
Q9Y6M1-1	556	320	236	0.58	0.42
Q96LT9	517	297	220	0.57	0.43
Q8NDT2	890	511	379	0.57	0.43
P29558	406	233	173	0.57	0.43

Q13283	466	267	199	0.57	0.43
Q13243	272	155	117	0.57	0.43
Q16560-2	251	143	108	0.57	0.43
B9ZVT1	881	501	380	0.57	0.43
Q9NTZ6	932	529	403	0.57	0.43
E2PSN0	171	97	74	0.57	0.43
Q14103	355	201	154	0.57	0.43
Q9BZC1-5	448	253	195	0.56	0.44
Q9NW13	759	427	332	0.56	0.44
Q96DH6	328	184	144	0.56	0.44
Q8N6W0	485	272	213	0.56	0.44
Q8N9W6-2	339	189	150	0.56	0.44
G5EA30	514	286	228	0.56	0.44
B0QYY7	200	111	89	0.56	0.45
Q9UN86-2	449	248	201	0.55	0.45
D6RIA2	172	95	77	0.55	0.45
P98179	157	86	71	0.55	0.45
Q9NVM6	304	166	138	0.55	0.45
Q9NQZ3	744	405	339	0.54	0.46
F8WE16	114	62	52	0.54	0.46
E9PC62	521	283	238	0.54	0.46
F8VZG9	466	253	213	0.54	0.46
F5GWN9	413	223	190	0.54	0.46
C9J2Z9	247	133	114	0.54	0.46
Q9NZI8	577	310	267	0.54	0.46
Q4G0J3	582	312	270	0.54	0.46
B7Z2K5	376	201	175	0.53	0.47
E7EWI9	322	172	150	0.53	0.47
C9JAA9	283	151	132	0.53	0.47
C9JYS8	248	132	116	0.53	0.47
Q9Y4C8	960	500	460	0.52	0.48
Q99729-2	332	172	160	0.52	0.48
F8W0K0	170	88	82	0.52	0.48
F6WRY4	373	193	180	0.52	0.48
H0Y623	466	241	225	0.52	0.48
Q9BX46	236	122	114	0.52	0.48
Q6ZRY4	209	108	101	0.52	0.48
J3KN81	165	85	80	0.52	0.48
H7C367	235	121	114	0.51	0.49
P31942	346	178	168	0.51	0.49
O14979	420	216	204	0.51	0.49
P78332	1123	576	547	0.51	0.49
F8W930	605	310	295	0.51	0.49
Q14498	530	271	259	0.51	0.49
Q92843-2	333	170	163	0.51	0.49
Q8IUH3	476	243	233	0.51	0.49

Q9H0Z9	239	122	117	0.51	0.49
Q5SZQ7	415	211	204	0.51	0.49
H7C476	135	68	67	0.50	0.50
O00425	579	291	288	0.50	0.50
Q8WUA2	492	246	246	0.50	0.50
Q13148-2	416	208	208	0.50	0.50
Q13148-3	260	130	130	0.50	0.50
H0YA61	80	40	40	0.50	0.50
Q9Y5S9	174	86	88	0.49	0.51
F8WD91	87	43	44	0.49	0.51
P52298	156	77	79	0.49	0.51
A6PVI3	153	75	78	0.49	0.51
E5RJV8	149	73	76	0.49	0.51
Q02040-3	445	216	229	0.49	0.51
F8WB35	200	97	103	0.49	0.52
C9JT33	438	212	226	0.48	0.52
H0Y5F5	550	266	284	0.48	0.52
H3BUA9	588	282	306	0.48	0.52
Q99729-4	286	137	149	0.48	0.52
H0Y4X3	337	160	177	0.47	0.53
F8W1T6	344	163	181	0.47	0.53
H0Y8T4	334	158	176	0.47	0.53
Q99729	332	157	175	0.47	0.53
G8JLB6	472	223	249	0.47	0.53
O60506	623	294	329	0.47	0.53
A0AV96	593	279	314	0.47	0.53
Q13151	305	143	162	0.47	0.53
Q32P51	320	150	170	0.47	0.53
Q13310-3	660	309	351	0.47	0.53
H0YIL2	110	51	59	0.46	0.54
O95758-4	558	258	300	0.46	0.54
F6T1J1	158	73	85	0.46	0.54
H0YCP8	273	126	147	0.46	0.54
P26368	475	219	256	0.46	0.54
E9PB51	240	110	130	0.46	0.54
Q13310	644	295	349	0.46	0.54
Q9UHX1	559	255	304	0.46	0.54
Q5H918	244	111	133	0.45	0.55
O43390-2	636	289	347	0.45	0.55
G3V2S9	124	56	68	0.45	0.55
P11940	636	285	351	0.45	0.55
Q17RY0-3	322	144	178	0.45	0.55
B4DMM2	186	83	103	0.45	0.55
Q13310-2	631	280	351	0.44	0.56
E5RFP2	339	150	189	0.44	0.56
B1ANR0	615	271	344	0.44	0.56

Q9H361	631	278	353	0.44	0.56
F8VRS4	391	172	219	0.44	0.56
P14866	589	259	330	0.44	0.56
B1APY8	385	169	216	0.44	0.56
H0YHU8	144	63	81	0.44	0.56
P09012	282	123	159	0.44	0.56
P52597	415	180	235	0.43	0.57
J3KP15	134	58	76	0.43	0.57
Q9Y3B4	125	54	71	0.43	0.57
P55795	449	192	257	0.43	0.57
Q4VXU2	614	262	352	0.43	0.57
E9PCY7	429	183	246	0.43	0.57
O14979-3	244	104	140	0.43	0.57
C9IYN3	282	119	163	0.42	0.58
F6W1U9	147	62	85	0.42	0.58
P31483-2	375	158	217	0.42	0.58
J3QL05	130	54	76	0.42	0.58
Q9UHX1-2	542	225	317	0.42	0.58
J3KQV8	1612	668	944	0.41	0.59
Q14576	367	152	215	0.41	0.59
F6UBL3	133	55	78	0.41	0.59
F5H0R1	912	376	536	0.41	0.59
F8W646	156	63	93	0.40	0.60
H0YJU7	72	29	43	0.40	0.60
P26599-3	557	224	333	0.40	0.60
J3KPK1	1526	610	916	0.40	0.60
B4DSS8	548	219	329	0.40	0.60
Q13242	221	87	134	0.39	0.61
Q01085-2	392	154	238	0.39	0.61
P26599	531	208	323	0.39	0.61
P26378-3	383	150	233	0.39	0.61
Q6ZP01	1051	410	641	0.39	0.61
P31483	386	149	237	0.39	0.61
D6W592	542	208	334	0.38	0.62
H0YEY9	128	49	79	0.38	0.62
Q12849	480	183	297	0.38	0.62
O75821	320	121	199	0.38	0.62
B7ZKM0	927	344	583	0.37	0.63
P05455	408	151	257	0.37	0.63
B1AM49	387	143	244	0.37	0.63
Q9H6T0	727	268	459	0.37	0.63
E9PKU1	114	42	72	0.37	0.63
Q5SZ64	190	69	121	0.36	0.64
C9JZG1	302	109	193	0.36	0.64
B4DTC1	131	47	84	0.36	0.64
H0Y3K3	84	30	54	0.36	0.64

D6RBK5	121	43	78	0.36	0.64
H0Y5U7	297	104	193	0.35	0.65
P08621-3	166	58	108	0.35	0.65
Q15020	963	335	628	0.35	0.65
Q9NQ94	594	206	388	0.35	0.65
O43251-3	358	122	236	0.34	0.66
C9J6C5	166	56	110	0.34	0.66
E9PIA5	39	13	26	0.33	0.67
H0YBN4	175	57	118	0.33	0.67
Q9Y4F3	1742	567	1175	0.33	0.67
B4DVB8	353	114	239	0.32	0.68
Q01081	240	77	163	0.32	0.68
Q8TBY0	533	169	364	0.32	0.68
H7BY16	297	94	203	0.32	0.68
D6RBM0	212	67	145	0.32	0.68
D6RF41	133	42	91	0.32	0.68
Q6NXG1	681	213	468	0.31	0.69
Q8N7X1	1067	333	734	0.31	0.69
E5RGV0	155	48	107	0.31	0.69
Q13247-2	135	41	94	0.30	0.70
Q8WU68-2	202	61	141	0.30	0.70
Q8IYX4	353	106	247	0.30	0.70
B8ZZ74	159	47	112	0.30	0.70
A6NLN1	197	58	139	0.29	0.71
C9JKX0	1430	421	1009	0.29	0.71
P08579	225	66	159	0.29	0.71
B4DUA4	138	40	98	0.29	0.71
C9JB16	90	26	64	0.29	0.71
E9PFH8	361	103	258	0.29	0.71
F8VV04	771	218	553	0.28	0.72
Q96IC2	774	217	557	0.28	0.72
H7BZE0	73	20	53	0.27	0.73
Q96IC2-2	743	203	540	0.27	0.73
E5RFV3	132	36	96	0.27	0.73
G8JLP4	1577	430	1147	0.27	0.73
O95453	639	171	468	0.27	0.73
H0YAP2	131	35	96	0.27	0.73
Q07955-2	292	77	215	0.26	0.74
G3V546	115	30	85	0.26	0.74
Q13287	307	79	228	0.26	0.74
J3QQZ2	328	83	245	0.25	0.75
J3QKT5	99	25	74	0.25	0.75
P38159-3	196	49	147	0.25	0.75
P55884-2	873	217	656	0.25	0.75
F8VTQ5	145	36	109	0.25	0.75
Q9P1R7	145	36	109	0.25	0.75

E5RG71	85	20	65	0.24	0.76
E7EPF2	128	30	98	0.23	0.77
J3KSW7	73	17	56	0.23	0.77
Q8WU68-3	181	42	139	0.23	0.77
C9JE21	204	47	157	0.23	0.77
Q5QPM2	89	20	69	0.22	0.78
E9PAM1	383	82	301	0.21	0.79
Q5T0W7	201	43	158	0.21	0.79
Q5TGA3	314	67	247	0.21	0.79
Q8WU68	220	46	174	0.21	0.79
Q5VZ19	366	76	290	0.21	0.79
Q96DU9	382	78	304	0.20	0.80
F8VYN5	113	22	91	0.19	0.81
H0YAM1	114	22	92	0.19	0.81
H0YCC8	197	38	159	0.19	0.81
E7ETC0	135	26	109	0.19	0.81
J3KTH5	428	82	346	0.19	0.81
H0YIB4	110	21	89	0.19	0.81
F2Z2W7	643	111	532	0.17	0.83
Q9NX07	287	49	238	0.17	0.83
Q8IZ69	625	106	519	0.17	0.83
H3BNC1	37	6	31	0.16	0.84
F8WD15	81	12	69	0.15	0.85
Q8TAS1	419	61	358	0.15	0.85
E5RG67	161	22	139	0.14	0.86
I3L0J9	1013	138	875	0.14	0.86
H3BR27	78	10	68	0.13	0.87
H0YJ07	56	7	49	0.13	0.88
F5H674	70	8	62	0.11	0.89
Q5JQF8	200	20	180	0.10	0.90
F2Z2U1	81	8	73	0.10	0.90
C9J787	92	9	83	0.10	0.90
B1ANR1	129	12	117	0.09	0.91
Q6P2Q9	2335	194	2141	0.08	0.92
G3V2B4	51	4	47	0.08	0.92
J3QKU3	65	5	60	0.08	0.92
E9PGX9	120	8	112	0.07	0.93
E5RI26	94	5	89	0.05	0.95
E5RJB9	129	6	123	0.05	0.95
